# An ST131 clade and a phylogroup A clade bearing an O101-like O-antigen cluster predominate among bloodstream *Escherichia coli* isolates from South-West Nigeria hospitals

**DOI:** 10.1099/mgen.0.000863

**Published:** 2022-12-16

**Authors:** Ayorinde O. Afolayan, A. Oladipo Aboderin, Anderson O. Oaikhena, Erkison Ewomazino Odih, Veronica O. Ogunleye, Adeyemi T. Adeyemo, Abolaji T. Adeyemo, Oyeniyi S. Bejide, Anthony Underwood, Silvia Argimón, Monica Abrudan, Abiodun Egwuenu, Chikwe Ihekweazu, David M. Aanensen, Iruka N. Okeke

**Affiliations:** ^1^​ Global Health Research Unit for the Genomic Surveillance of Antimicrobial Resistance, Department of Pharmaceutical Microbiology, Faculty of Pharmacy, University of Ibadan, Oyo State, Nigeria; ^2^​ Department of Medical Microbiology and Parasitology, Obafemi Awolowo University Teaching Hospitals Complex, Ile-Ife, Osun State, Nigeria; ^3^​ Department of Medical Microbiology and Parasitology, University College Hospital, Ibadan, Oyo State, Nigeria; ^4^​ Department of Medical Microbiology and Parasitology, Obafemi Awolowo University Teaching Hospitals Complex, Ile-Ife, Osun State, Nigeria; ^5^​ Department of Medical Microbiology and Parasitology, University of Osun Teaching Hospital, Osogbo, Osun State, Nigeria; ^6^​ Centre for Genomic Pathogen Surveillance, Big Data Institute, University of Oxford, Old Road Campus, Oxford, UK; ^7^​ Wellcome Genome Campus, Hinxton, UK; ^8^​ Nigeria Centre for Disease Control, Jabi, Abuja, Nigeria

**Keywords:** antimicrobial resistance, *cpsACP*, *Escherichia coli*, genomic surveillance, Nigeria, ST131

## Abstract

*
Escherichia coli* bloodstream infections are typically attributed to a limited number of lineages that carry virulence factors associated with invasiveness. In Nigeria, the identity of circulating clones is largely unknown and surveillance of their antimicrobial resistance has been limited. We verified and whole-genome sequenced 68 2016–2018 bloodstream *E. coli* isolates from three sentinel sites in South-Western Nigeria and susceptibility tested 67 of them. Resistance to antimicrobials commonly used in Nigeria was high, with 67 (100 %), 62 (92.5 %), 53 (79.1 %) and 37 (55.2 %) showing resistance to trimethoprim, ampicillin, ciprofloxacin and aminoglycosides, respectively. Thirty-five (51 %) isolates carried extended-spectrum β-lactamase genes and 32 (91 %) of these were multidrug resistant. All the isolates were susceptible to carbapenems and colistin. The strain set included globally disseminated high-risk clones from sequence type (ST)12 (2), ST131 (12) and ST648 (4). Twenty-three (33.8 %) of the isolates clustered within two clades. The first of these consisted of ST131 strains, comprising O16:H5 and O25:H4 sub-lineages. The second was an ST10–ST167 complex clade comprising strains carrying O-antigen and capsular genes of likely *Klebsiella* origin, identical to those of avian pathogenic *E. coli* Sanji, and serotyped *in silico* as O89, O101 or ONovel32, depending on the tool used. Four temporally associated ST90 strains from one sentinel were closely related enough to suggest that at least some of them represented a retrospectively detected outbreak cluster. Our data implicate a broad repertoire of *E. coli* isolates associated with bloodstream infections in South-West Nigeria. Continued genomic surveillance is valuable for tracking clones of importance and for outbreak identification.

## Data Summary

Phylogenetic tree, clinical data and epidemiological data were visualized using Microreact (https://microreact.org/project/hmj3KwxS1dmmFPCKFx6qeA-invasive-escherichia-coli-sw-nigeria-2016-2018). All the sequence data have been deposited in the ENA (European Nucleotide Archive) under the project ID PRJEB29739 (https://www.ebi.ac.uk/ena/browser/view/PRJEB29739). Accession numbers can be found in Table S1, available with the online version of this article. Genome assemblies can be found in Pathogenwatch using the following links (https://pathogen.watch/collection/aku4gm6a16gq-bloodstream-escherichia-coli-in-south-west-nigerian-hospitals-20; https://pathogen.watch/collection/0f6pm7vqfzjs-bloodstream-escherichia-coli-in-south-west-nigerian-hospitals-20; and https://pathogen.watch/collection/423wubi6peru-bloodstream-escherichia-coli-in-south-west-nigerian-hospitals-20).

**Impact Statement**

*
Escherichia coli* bloodstream infections are serious and potentially fatal. Managing and containing these infections is improved when health systems have information about which lineages prevail in specific locations, what disease-causing attributes they have, and which drugs can be used to eliminate them. Very little data along these lines are available in Nigeria. We tested the antimicrobial susceptibility of 68 bloodstream *E. coli* isolates from three Nigerian hospitals and used whole-genome sequencing (WGS) to identify and characterize lineages of concern. Globally disseminated, high-risk lineages were detected among the isolates, one of the most abundant being sequence type 131, which has been reported from around the world. We additionally identified much less well known genetically related strains with surface features that are likely to have been acquired from *Klebsiella* bacteria and require further characterization. A retrospectively detected outbreak caused by a less-abundant lineage was also uncovered using the sequence data. These and other lineages carried a broad repertoire of antimicrobial-resistance genes, including those conferring resistance to extended-spectrum cephalosporin drugs that are critical for managing these infections in Nigeria. Overall, the study demonstrated the utility and value of WGS for identifying important lineages and possible outbreaks due to *E. coli* in Nigeria.

## Introduction

Extra-intestinal pathogenic *Escherichia coli* (ExPEC) are the most commonly recovered bacterial isolates from infections in the blood, urine, meninges, prostate and other normally sterile sites [1–3]. Although typically initially acquired within the gastrointestinal tract (and less commonly via the genital route), these strains of *E. coli* differ from commensal and diarrhoeagenic *E. coli* in possession of factors associated with systemic virulence [4, 5], allowing them to survive in different extra-intestinal niches. Some of the virulence genes associated with ExPEC include adhesins (*fim*, *pap*, *sfa*, *afa*), invasins (*ibeA*), iron acquisition genes (*ybt*, *iro*, *iuc*), toxins (*clb*, *cnf*, *hly*, SPATE genes) and protectins (*traT*, *ompT*, *kpsMT*), among others [4, 5]. ExPEC also often have K, O and H antigens that make them recognizable to *E. coli* experts and, in some instances, assist them in evading immune responses. Virulence and antimicrobial-resistance (AMR) determinants, as well as negative frequency-dependent selection, likely influence the stability of the most dominant ExPEC groups, which are often multiply resistant [6], thereby sustaining the occurrence of extra-intestinal diseases globally. In Africa, available data reveal that there is an upward trend in the prevalence of globally dominant ExPEC lineages in humans [7] and animals [8], painting a grim picture for disease and AMR.

Robust surveillance is urgently needed to tackle AMR in a more responsive and consistent manner within each country. Whole-genome sequencing (WGS) has helped to provide a clearer picture on the epidemiology of infectious diseases caused by ExPEC and has identified a number of pandemic clones. Incorporation of WGS in existing epidemiological frameworks of national public-health institutes is critical for providing genomic context for prospective surveillance and for designing and implementing AMR-eliminating strategies.

Although studies conducted in Africa and other low- and middle-income countries (LMICs) have shown the abundance of *E. coli* in infections from normally sterile sites [9, 10], these studies are few and far between, so that ExPEC and their AMR epidemiology are poorly understood [7, 11]. In Nigeria, there is sparse molecular information on ExPEC, but a few studies point to likely clonal expansion of resistant lineages and local presence of pandemic clones of concern [12–16]. These studies provide valuable information but represent an insufficient picture of ExPEC clones in Nigeria, with little data available from the South. To extend the body of knowledge on genomic epidemiology of ExPEC in South-Western Nigeria, including AMR patterns and mechanisms, we leveraged on our recently established genomic surveillance of bacterial AMR efforts by characterizing the genomes of bloodstream isolates from tertiary hospitals in South-West Nigeria.

## Methods

### Species validation and antimicrobial-susceptibility tests (ASTs)

Sentinel hospital laboratories referred anonymized clinical data and bloodstream *E. coli* isolates collected between the years 2016 and 2018 to our National Reference Laboratory, University College Hospital, Ibadan, Nigeria for verification of isolate identity and ASTs. Isolate identity and ASTs were achieved using the VITEK 2 instrument. Drugs tested include amikacin (AMK), gentamicin (GEN), ampicillin (AMP), amoxicillin/clavulanic acid (AMC), piperacillin/tazobactam (TZP), cefuroxime (CXM), cefuroxime axetil (CXMA), cefepime (FEP), ceftriaxone (CRO), cefoperazone/sulbactam (SFP), nitrofurantoin (NIT), nalidixic acid (NAL), ciprofloxacin (CIP), ertapenem (ERT), imipenem (IPM), meropenem (MEM) and trimethoprim/sulfamethoxazole (SXT). Resistance profiles were generated from VITEK 2 AST data. Antibiotic-susceptibility results were interpreted in line with the CLSI (Clinical and Laboratory Standards Institute) 2019 standards [17]. Minimum inhibitory concentration values were converted to Reisistant-Intermiediate-SSensitive (RIS) designations and the bug-drug combination table was generated using the AMR R package (v1.4.0; https://github.com/msberends/AMR) [18].

### Biofilm assay

Following Wakimoto *et al*’s (2004) procedure [19], we sub-cultured bloodstream *E. coli* isolates overnight in LB broth at 37 °C with shaking at 160 r.p.m. Afterwards, we measured 190 µl Dulbecco’s modified Eagle’s medium (DMEM) containing 0.45 % glucose using a pipette into each well of a 96-well plate [different plates were used for each time point (3, 6, 12, 24 h)]. Five microlitres of each isolate was inoculated in triplicate per time point into a 96-well plate from the stock plate and incubated at 37 °C without shaking until each time point was reached. Absorbance at 595 nm was measured on completion of the time point.

We pipetted spent media out of the 96-well plates. Each well was washed with PBS three times, fixed (10 mins, 200 µl 75% ethanol), dried and stained with 195 µl 0.5% crystal violet for 5 min. This was followed by washing and drying of the plates. We added 200 µl 95% ethanol to each well, allowed the wells to stand for 20 min at room temperature, and determined absorbance with an ELISA plate reader at 570 nm. Biofilm index was defined using the mean of the values for the absorbance at 570 and 595 nm, and was calculated by dividing the absorbance values for each strain at a given time point by the absorbance values of the negative control at the given time point [20]. Enteroaggregative *E. coli* strain 042 was used as a positive control, while *E. coli* K-12 DH5α was used as negative control. The magnitude of biofilm formed by different genetically defined subgroups of isolates was compared using a two-tailed Mann–Whitney test at *P* <0.05.

### Haemolysis test

Overnight LB cultures of the isolates were spotted onto the surface of blood agar. α-Haemolysis and β-haemolysis is indicated by a green colouration and a clear zone around bacterial colonies, respectively. Uropathogenic *E. coli* isolate ATCC 11175 and *stx*-deleted *E. coli* O157 isolate ATCC 7000728 were used as controls.

### DNA extraction, library preparation and WGS

Genomic DNA was extracted following the protocol outlined in a previous report [21]. Briefly, the Wizard DNA extraction kit (Promega) was used in the extraction of genomic DNA of all isolates. A dsDNA broad range quantification assay was used in the quantification of DNA extracts (Invitrogen). DNA libraries were prepared and sequenced using the NEBNext Ultra II FS DNA library kit (New England Biolabs) and HiSeq X10 instrument (Illumina), respectively.

### Genome assembly and speciation

Raw sequence reads were assembled using the GHRU (Global Health Research Unit) for genomic surveillance of antimicrobial resistance pipeline (https://gitlab.com/cgps/ghru/pipelines/assembly), which is summarily explained by the GHRU *de novo* assembly protocol [22]. Speciation was possible using BactInspector (v0.1.3; https://gitlab.com/antunderwood/bactinspector/), implemented within the GHRU pipeline.

### SNP analysis and phylogenetic tree generation

The complete chromosome sequence of *E. coli* strain EC958 (accession no. GCF_000285655.3) (https://www.ncbi.nlm.nih.gov/assembly/GCF_000285655.3/) was used to infer a whole-genome alignment of the sequence reads and identify SNP positions, which were, in turn, used to infer a maximum-likelihood phylogenetic tree as per the GHRU mapping-based phylogeny protocol [22], which summarizes the steps implemented within the GHRU SNP phylogeny pipeline (https://gitlab.com/cgps/ghru/pipelines/snp_phylogeny).

Isolates that clustered closely within the phylogenetic tree, belonged to the same sequence type (ST), and shared similar resistance profiles, plasmid profiles, geography and dates of isolation, were investigated further for clues on potential outbreaks. We selected the closest reference genome to the outbreak isolates using BactInspector, aligned the likely outbreak isolates (ST90) to the reference genome of *E. coli* strain D3 (accession no. NZ_CP010140.1; https://www.ncbi.nlm.nih.gov/assembly/GCF_001900635.1/), used Gubbins [23] to mask recombinant regions, and calculated pairwise SNP distances from the pseudo-genome alignment using FastaDist (v0.0.7; https://gitlab.com/antunderwood/fastadist), SNP-dists (v0.8.2; https://github.com/tseemann/snp-dists) and/or the R package harrietr (v0.2.4; https://github.com/andersgs/harrietr). The adegenet R package (v2.1.5; https://github.com/thibautjombart/adegenet/) was used to generate a pseudogenome alignment in DNA.bin format (one of the input files accepted by harrietr) from a pseudogenome alignment file. Phylogenetic tree, epidemiological data and *in silico* data were visualized in the web-based viewers Microreact [24] and the Interactive Tree of Life (iTOL) [25].

### AMR gene, virulence gene and plasmid replicon prediction

The program srst2 (v0.2.0; https://github.com/katholt/srst2/) [26] and the ARGannot database (https://raw.githubusercontent.com/katholt/srst2/master/data/ARGannot_r3.fasta) were used to screen raw sequence reads for the presence of acquired resistance genes. We also validated the report/output by utilizing the GHRU AMR Pipeline (https://gitlab.com/cgps/ghru/pipelines/dsl2/pipelines/amr_prediction), as explained in this protocol [22]. *ampC1* and *ampC2* were excluded from downstream analysis and visualization as they are β-lactamase genes present in almost all *E. coli* isolates and are unlikely to confer antibiotic resistance in *E. coli* [27]. An overall plot of resistance determinants was constructed using the *upset* function from the UpSetR package (v1.4.0) [28]. Plots of AMR genes stratified by ST and sentinel site were constructed using the ggupset package (v0.3.0). Association between virulence genes and phylogroups of *E. coli* was determined using the *fisher_test* function from the rstatix package (v0.7.0). The calculation is based on single genes (including those that make up an operon), not operons. The associations were corrected for multiple testing using the Bonferroni method offered by rstatix. Bar plots were visualized using the *ggplot* function from the Tidyverse package (v1.3.1) in R.

The raw reads were screened for virulence genes with the GHRU pipeline, which utilizes ariba [29] and the VFDB database [30]. Plasmid replicon types were determined with same GHRU pipeline but using the PlasmidFinder database instead [31].

### Multilocus sequence typing (MLST) profiling

Following the Achtman MLST scheme [32], multilocus STs were determined using the srst2 program. We confirmed the results by using the GHRU MLST pipeline, which also implements the Achtman scheme. as summarized in the aforementioned GHRU protocol [22].

### *In silico* serotyping, phylogroup determination and *fimH* typing

The O- and H- serotypes of *E. coli* were determined using the srst2 program and the EcOH database (https://raw.githubusercontent.com/katholt/srst2/master/data/EcOH.fasta). This result was cross-checked using ECTyper (v1.0.0; https://github.com/phac-nml/ecoli_serotyping) and SerotypeFinder (https://bitbucket.org/genomicepidemiology/serotypefinder/src/master/). SerotypeFinder utilizes kma [33] and blast+ [34] to predict *E. coli* serotypes from the alignment of raw and assembled reads, respectively, against the SerotypeFinder database. Because the srst2-predicted O serotype Onovel32 was predicted differently by the *in silico* tools ECTyper and SerotypeFinder as O89 and O101, respectively, these O antigen gene clusters were extracted from their respective databases (srst2, ECTyper, SerotypeFinder) and were aligned using muscle with default settings within mega x (v10.2.6) [35]. A maximum-likelihood phylogenetic tree was recreated from the alignment using mega x.

*E. coli* genomes were assigned into phylogroups using ClermonTyping (v20.03) [36], and were subtyped based on *fimH* alleles, using the FimTyper command-line tool (https://bitbucket.org/genomicepidemiology/fimtyper/src/master/) [37].

### Concordance

The agreement between phenotypic and genotypic AMR was determined for β-lactams, cephalosporins, amikacin/kanamycin/gentamicin, trimethoprim and the quinolones. Metrics such as sensitivity, specificity, true positives, true negatives, false positives and false negatives were determined using the R script (https://gitlab.com/-/snippets/2050300, first used in a previous report), which utilizes the *epi.tests* function within the epiR package (v2.0.26) for each antimicrobial tested. Here, ‘sensitive’ and ‘intermediate’ values were combined, taking cognizance of the arguments for and against the use of the term intermediate in clinical settings [38].

### Genome annotation, comparative genomics and gene location prediction

Functional annotation of Onovel32 clade genomes was performed using Bakta v1.0.4 [39] . Genomes were compared and visualized using Artemis v18.1.0 [40], Artemis Comparison Tool v18.1.0 [41] and Clinker v0.0.21 [42]. The mlplasmid web tool [43] was used to predict whether clinically relevant virulence genes were borne on plasmids or on chromosomes.

## Results

### Epidemiology and species identification

Three hospital laboratories in South-West Nigeria submitted retrospective bloodstream *E. coli* isolates with clinical and epidemiological data between the years 2016 and 2018. Available data showed that isolates were collected from patients aged 1 day to 71 years (male=20, female=24 – of those for which gender information is available; median age=37 years), with 17 (25 %) of the isolates recovered from children under 28 days old (range 1–20 days, median 9 days), who would be characterized as neonates. The isolates were submitted from the University College Hospital (UCH; *n*=22), Obafemi Awolowo University (OAU) Teaching Hospitals Complex (*n*=18) and Osun State University Teaching Hospital (until recently known as Ladoke Akintola University) (LAU; *n*=28).

Of the 68 bloodstream isolates confirmed as *E. coli* by WGS, 48 (70.6 %) and 64 (94.1 %) were correctly identified as *E. coli* by the sentinel biochemical testing and reference laboratory VITEK 2, respectively. *E. coli* isolates were often misidentified as *Klebsiella pneumoniae* (*n*=8) or *Citrobacter freundii* (*n*=6) at the sentinel laboratories, while the VITEK 2 system misidentified *E. coli* as *K. pneumoniae* (*n*=3) or *Enterobacter aerogenes* (*n*=1).

### Phylogroups, serotypes, STs and FimH types of *E. coli* bloodstream isolates

**Fig. 1.**
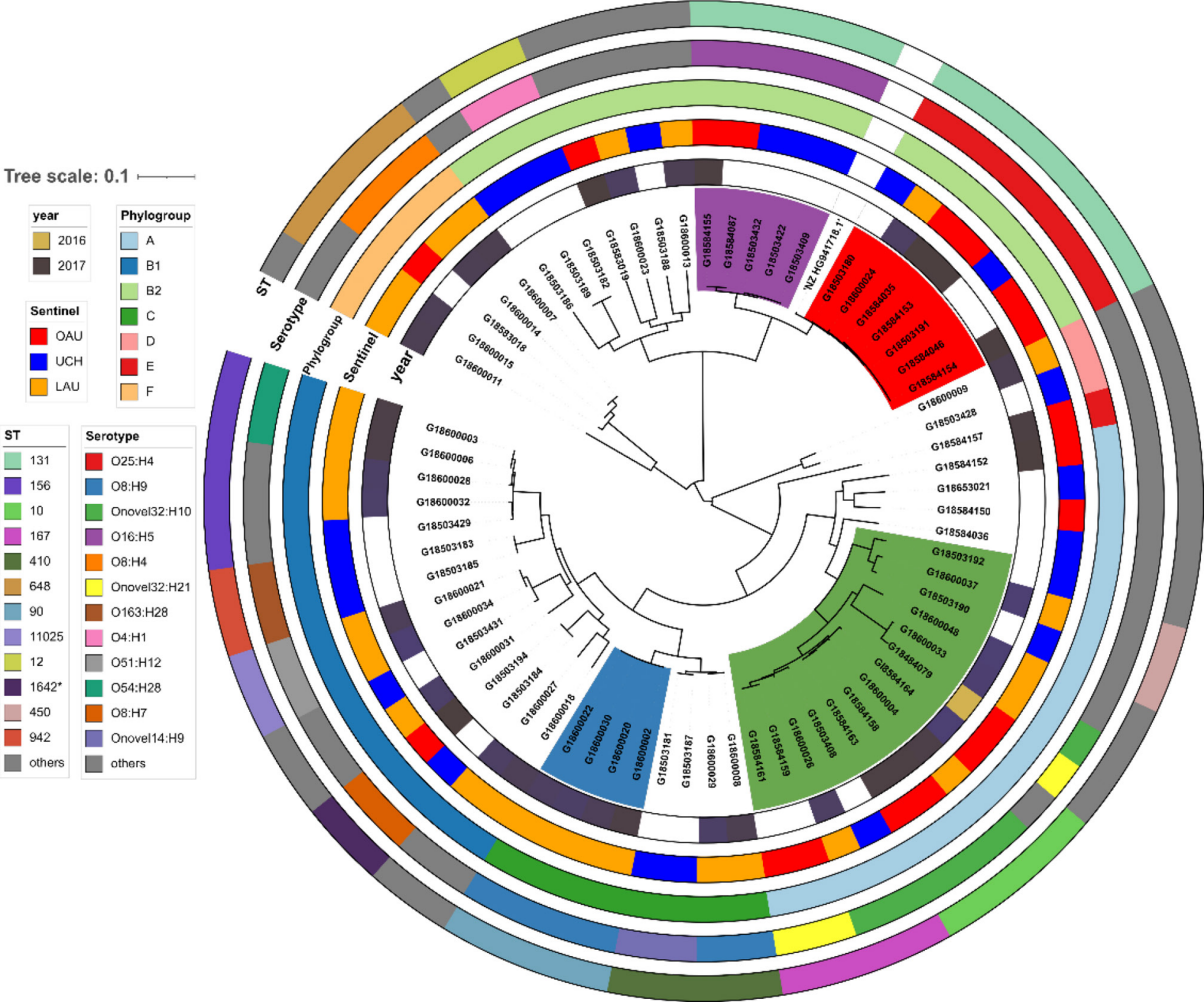
Maximum-likelihood SNP tree of bloodstream *E. coli* isolates sequenced for this study. The tree scale shows substitutions per site based on alignment length. The purple-coloured clade represents ST131 lineage 1 (O16:H5 serotype); the red clade represents the ST131 lineage 2 (O25:H4 serotype); the light green clade represents the ST10–ST167 clade. The light blue clade marks the cluster from a single site representing a likely outbreak clone of ST90.

*E. coli* sent from all three hospital sentinel laboratories spanned all *E. coli* phylogroups, with 18, 15, 19, 8, 2, 1 and 5 *E. coli* genomes classified within phylogroups A, B1, B2, C, D, E and F, respectively. The most common STs among the 33 identified include: ST131 (*n*=12), ST156 (*n*=5), and 4 each of ST10, ST167 ST410, ST648 and ST90. These seven STs accounted for 54 % of the *E. coli* isolates. Of these, only STs 131 and 167 were found across the three sentinel sites (Fig. 1, Table S1). Diverse lineages were recovered from all three sentinel sites (OAU=10 STs, LAU=18 STs, UCH=15 STs) (Fig. 1). While ST131 genomes made up 63% of genomes within phylogroup B2 (the second most common phylogroup), ST10 and ST167 genomes accounted for 44% of genomes within phylogroup A (the most common phylogroup).

While none of the isolates were submitted as suspected outbreak strains, four isolates recovered from LAU within 1 month (January 2017) were typed as ST90, serotype O8:H9, and carried the same plasmid profile (IncFI; IncFIA; IncFIB_AP001918; IncQ1), AMR gene profile, and similar but not identical resistance profile (Table S1). These four isolates were resistant to trimethoprim, the quinolones, gentamicin, cefuroxime axetil and ampicillin (Table S1). Comparison of pairwise SNP differences revealed that two of the four isolates were identical, while the other two isolates differed from these by 11 SNPs and 72 SNPs. The current literature supports a clustering threshold between the range of 0 to 17 SNPs for suspected outbreak-related *E. coli* blood isolates [44–46]. Although these isolates were correctly identified within this sentinel site, the cluster was only recognized retrospectively, likely due to different β-lactam resistance profiles.

ST131 was the most common ST detected and the 12 isolates belonging to this ST clustered into two distinct lineages defined by serotype H5 (*n*=5; O16:H5) and the serotype H4 lineage (*n*=7; O25:H4) and, henceforth, are referred to as the ST131 lineage 1 and ST131 lineage 2, respectively (Fig. 1).

A total of 38 unique serotypes and 25 O-groups were identified. Of note, Onovel32 was the most common O-type identified by srst2, which established the O-type as novel and identified it in 11 isolates belonging to phylogroup A. These ONovel:32 strains belonged to ST10 or ST167, or were single or double locus variants of these STs. They included six ONovel32:H10 isolates, as well as three H21 and one H4 flagellin-encoding strains. Along with one ONT:H10 strain that also belonged to ST10, they formed a distinct clade on the phylogenetic tree (Fig. 1). Strains belonging to this cluster were submitted from all three hospitals. Two of them were originally misclassified as *K. pneumoniae* by VITEK 2 at the reference laboratory level. Irrespective of whether the ST90 clade is discounted, the ONovel:32 clade and the ST131 clade were the most abundant. Together they accounted for 32.4% of the isolates and both clades were found in all three hospitals (Fig. 1).

A total of 20 unique FimH types were identified in 82% of ExPEC isolates (*n*=56/68), including *fimH*54 (*n*=10), *fimH*30 (*n*=8), fimH24 (*n*=5) and *fimH*41 (*n*=5) – the four most common FimH types observed. ST131 isolates carried *fimH*30 (*n*=7, ST131 lineage 2) and *fimH*41 (*n*=5, ST131 lineage 1) types, corresponding to the two unique ST131 lineages. Nine of the Onovel32 isolates belonged to the *fimH*54 type [ST10 (*n*=4), ST167 (*n*=4), ST44 (*n*=1); no *fimH* type was observed in the remaining two, that is, ST617 and ST1284 isolates] (Table S1). Overall, no *fimH* type was observed in isolates belonging to ST12 (*n*=2), ST648 (*n*=4) and six other STs (STs 46, 181*, 315, 617, 1284, 1808).

### Virulence factor profiles of the bloodstream *E. coli* isolates

**Fig. 2.**
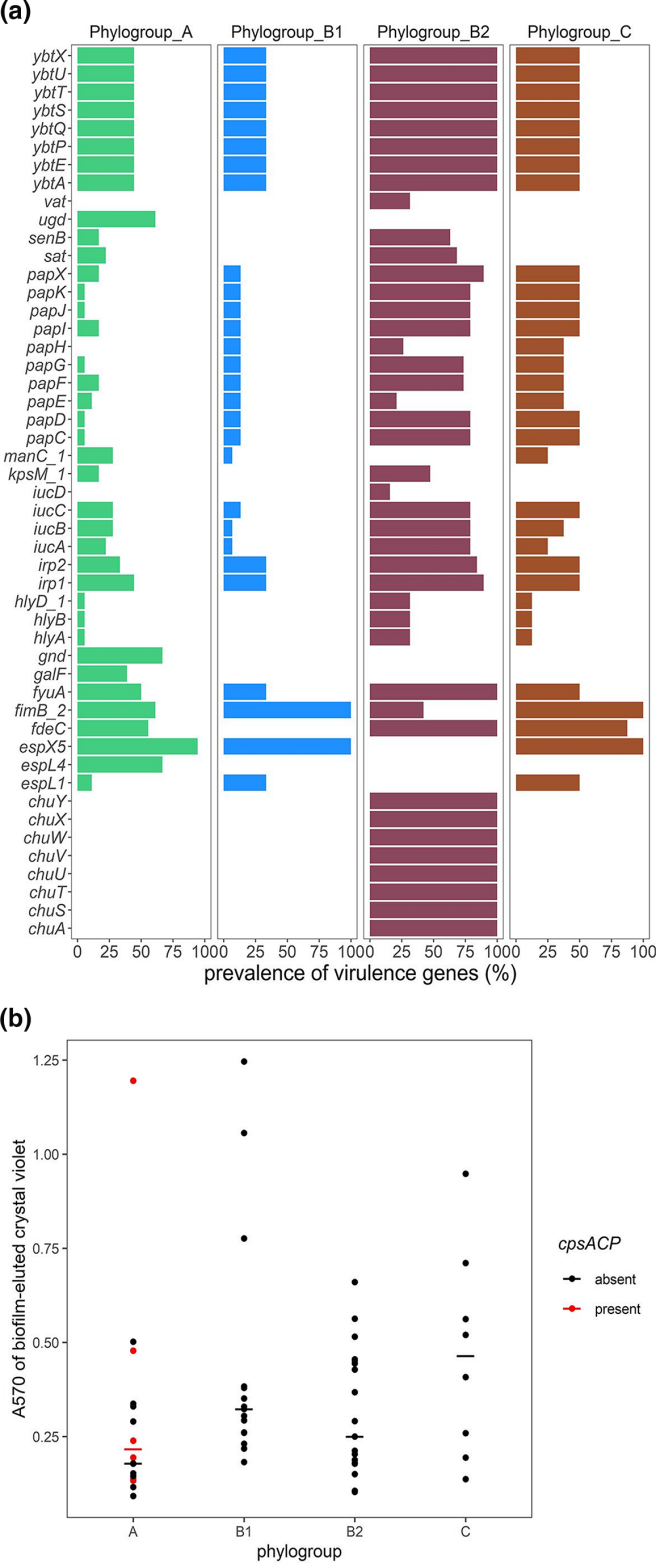
(a) Comparison of virulence genes among isolates clustered within phylogroups A–C. The graph shows the prevalence of virulence factors in isolates within phylogroups containing more than five isolates: A (*n*=18), phylogroup B1 (*n*=15), phylogroup B2 (*n*=19) and phylogroup C (*n*=8). Using Fisher’s test (*P*<0.05), only the VAGs significantly more prevalent in at least one phylogroup are shown. (b) Biofilm formation in 67 strains, measured as *A*_570_ of crystal violet eluted from fixed and stained 6 h biofilms. Each dot represents data from a single strain belonging to the phylogroup listed on the *x*-axis. Horizontal bars mark the median for each phylogroup, outliers inclusive due to the small number of tested strains in each phylogroup. The *A*_570_ values for isolates carrying the *cpsACP* gene are represented as red dots.

Diverse virulence genes were observed among the ExPEC genomes: 159 virulence-associated genes (VAGs) were found at least once in the 68 bloodstream isolate genomes (Table S2). Enterobactin genes (*entB*, *entC*, *entE_1*, *entS*) and ferrienterobactin precursors and proteins [*fep* operon genes (ABCDEG) and *fes_1*] were found in more than 95% of the isolates. The outer membrane hemin receptor (*chu*), siderophores (*fyuA*, *irp*, *ybt*), intimin-like adhesin (*fdeC*), haemolysin (*hly*), aerobactin (*iuc*)*,* polysialic acid transport protein *kpsM_1*, pyelonephritis-associated pili *pap*, SPATE genes (*sat*, *vat*) and plasmid-encoded *Shigella* enterotoxin *senB* were more abundant and significantly more associated with phylogroup B2 than with phylogroups A, B1 and C (Fisher’s exact test, *P* <0.05; Fig. 2a). Fig. 2(b) shows that a wide range of biofilm-forming capacities were seen in the subset of isolates tested, with moderate or strong biofilm-formers being most common in phylogroups B1 and B2 as well as the ST90 outbreak strain-containing phylogroup C. No significant difference in biofilm-formation between the phylogroups was observed.

**Fig. 3.**
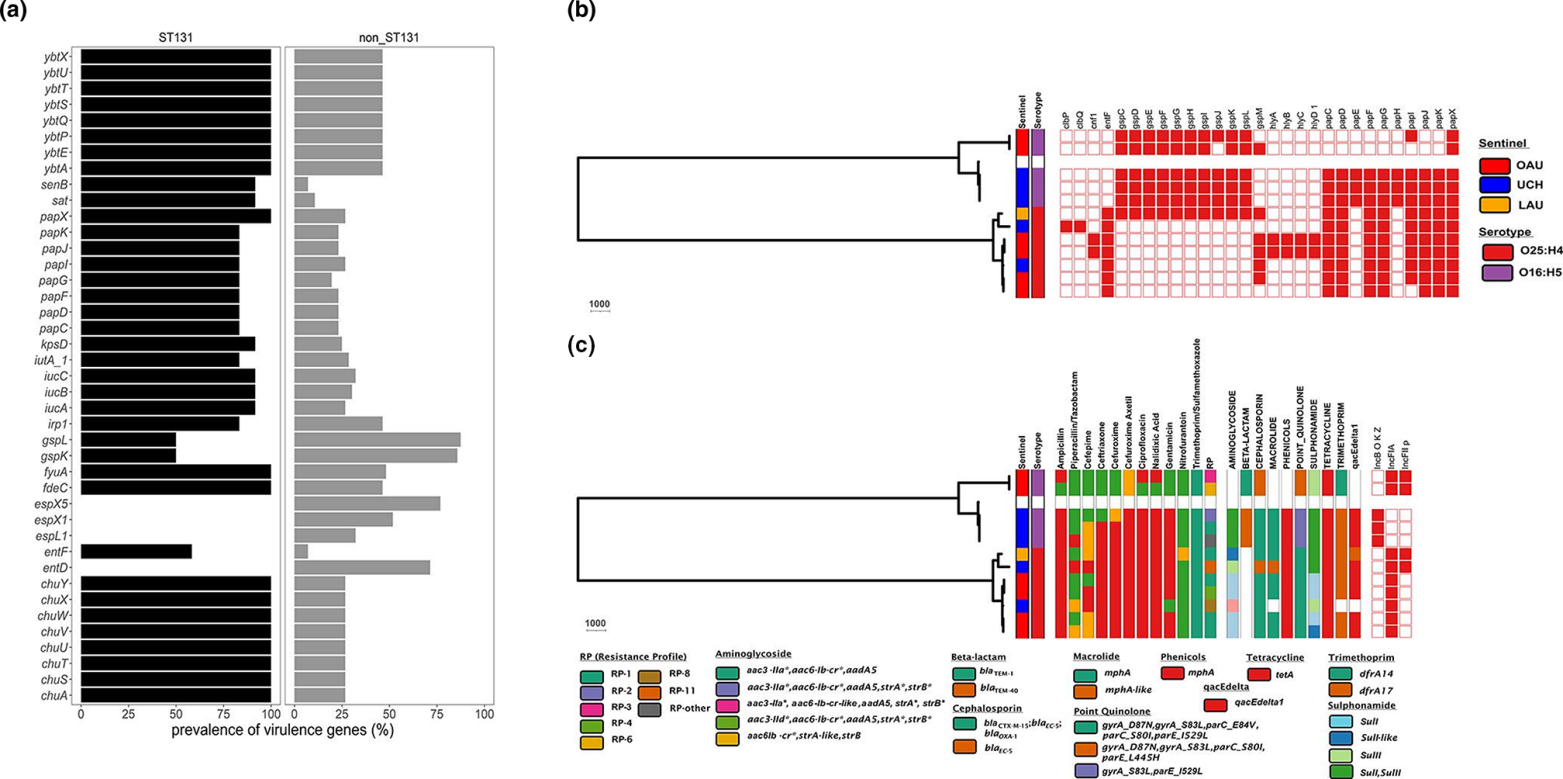
(a) Comparison of virulence genes between ST131 (*n*=12) and non-ST131 (*n*=56) isolates. Using Fisher’s test (*P*<0.05, Bonferroni corrected), only the VAGs significantly more prevalent in the STs are shown. (b) Virulence factor profile of isolates belonging to ST131. The presence or absence of a virulence gene is indicated by a red or white colour on the heatmap, respectively. (**c)** AMR phenotype profile and AMR genotype profile (uppercase headers, apart from *qacEdelta1*) of isolates belonging to ST131. The presence or absence of plasmid Inc types is indicated by a red or white colour on the heatmap, respectively. Tree scales in (b) and (c) show substitutions per site based on alignment length.

Phylogroup B2 isolates, comprised largely of ST131 strains, carried the highest number of VAGs (*n*=86). Thirty-four VAGs were significantly more common in ST131 isolates (*n*=12) than in non-ST131 isolates (*n*=56). Of these, the genes encoding the outer membrane haem receptor (*chu*), yersiniabactin (*ybt*) and pyelonephritis-associated pili (*pap*) were found in more than 84% of the ST131 genomes (Fisher’s exact Test, *P* <0.05; Fig. 3a). However, six VAGs were significantly more common in non-ST131 isolates than in ST131 isolates, including two genes (*gspK* and *gspL*) present in more than 85 % of the non-ST131 isolates, but in only about 50 % of ST131 isolates. The VAGs *entD*, *espL1*, *espX1* and *espL5* were absent in ST131 isolates but were present in at least 32 % of non-ST131 isolates, notably phylogroup A ONovel32 strains (Fig. 3a).

Both lineages of ST131 share common VAGs, including adhesins, yersiniabactin, aerobactin, enterotoxin and transport-associated genes, but some genes were only seen, or predominantly seen, in one of the two lineages. For example, the haemolysin (*hly*) carried by two isolates within ST131 lineage 2 were absent in ST131 lineage 1. Also, two isolates within ST131 lineage 2 carried the cytotoxic necrotizing factor *cnf1* (Fig. 3a, b). The median pairwise distance between isolates in ST131 lineage 1 is 2857.5 SNPs (range: 0–2866), while the median pairwise distance between isolates within ST131 lineage 2 is 499 SNPs (range: 0–827). The inter-clade SNP distance is 13 619 (range: 12900–14146) (Fig. S1). The two lineages of ST131 clade share 4116 core genes. On the one hand, isolates within the ST131 lineage 1 carried 121 accessory genes, including: mobile elements (IS*110* family transposase IS*Ec21*, insertion sequence IS*5376* putative ATP-binding protein, insC-1, *insB1*, *insCD1*, *ykfF*, *yjhR*_2, *ybcK*, *ybcQ*, umuD_2, *rrrD*), and genes involved in multi-drug efflux (*emrE*), DNA binding (*arcA*, *argR*_2), amino acid binding (argI_2), ATP binding (*htpG_2*, *idnK*), PTS permease activity (*frvA*, *frvB*, *sgcA*_1), SOS response (*umuC*_4, essD_2). On the other hand, isolates within the ST131 lineage 2 carried 284 accessory genes, including: prophage protein genes (*gpFI*_2), mobile elements (IS*4* family transposase IS*Ec13*, IS*200*/IS*605* family transposase IS*200C*, Tn*3* family transposase, intE_2, *insN2*, *intS*, *nohB*, *pine*, *rrQ*, *rzpD*, *ydfD*, *ynfO*), and genes involved in structural molecule activity (*flg*, *fli*). A greater proportion of hypothetical genes were identified in lineage 2 (~71%; *n*=202/284) than lineage 1 (~39%; *n*=47/121).

**Fig. 4.**
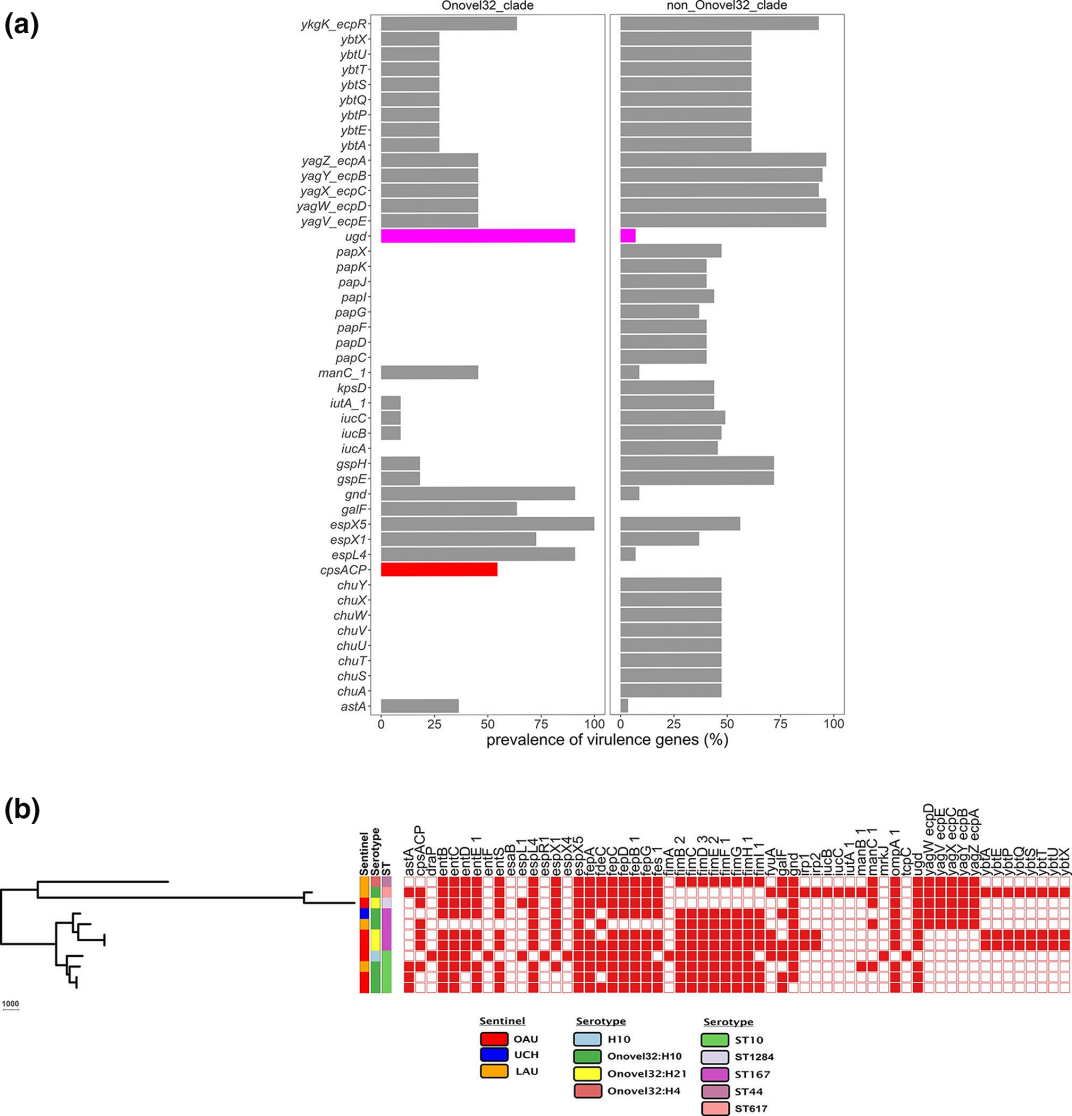
(a) Comparison of virulence genes between Onovel32 (*n*=11) and non-Onovel32 (*n*=57) clade isolates. Using Fisher’s test (*P* <0.05, Bonferroni corrected), only the virulence genes significantly more prevalent are shown. The *cpsACP* gene and the *ugd* gene bars are coloured red and magenta, respectively. (b) Onovel32 clade isolates belonging to the STs ST10, ST167 and their locus variants belong to phylogroup A. The presence or absence of a virulence gene is indicated by a red or white colour on the heatmap, respectively.

**Fig. 5.**
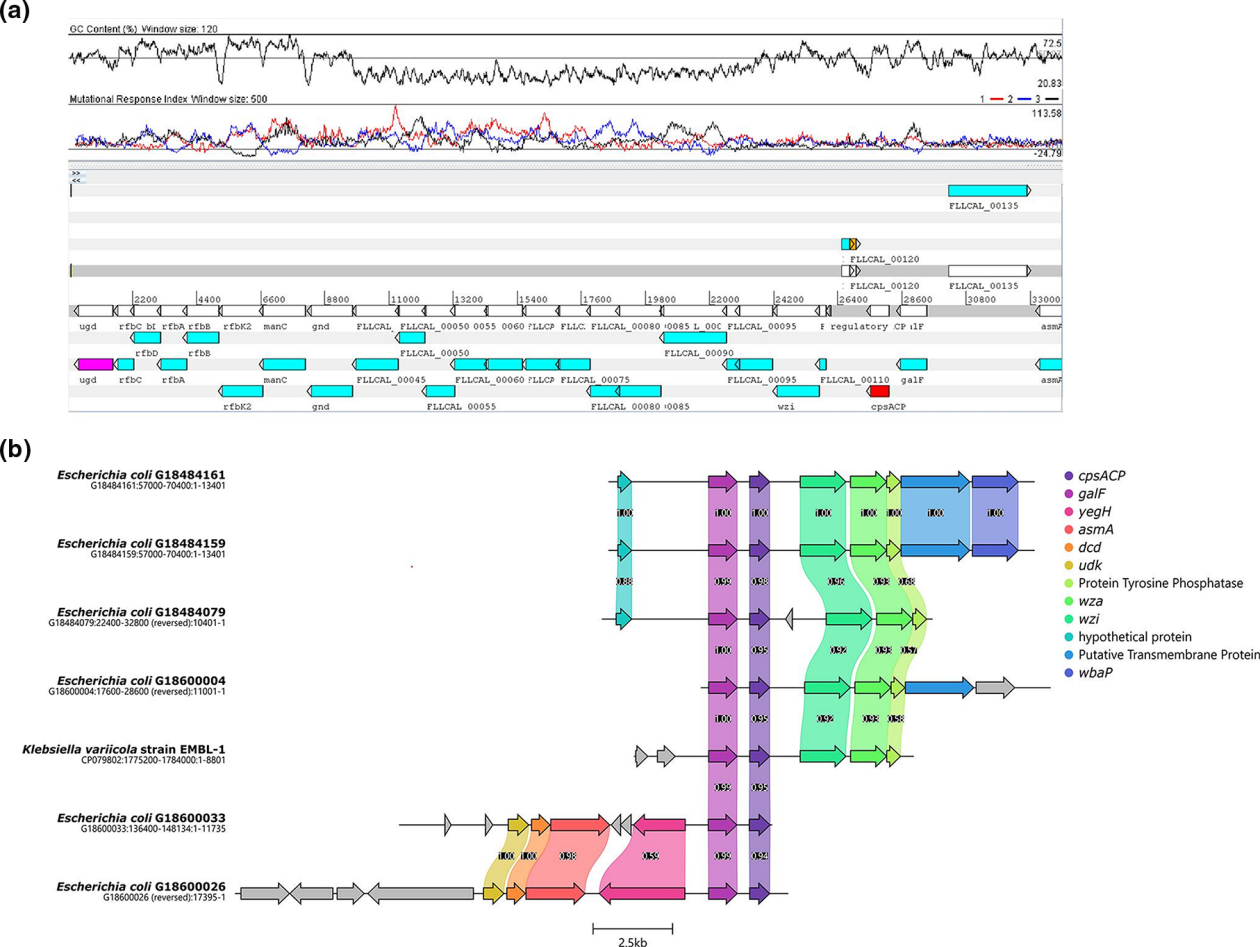
(a) Schematic cluster in ST1294 ONovel32 chromosome that includes the *ugd* and *cpsACP* genes. The genes within the cluster are syntenic and identical with a cluster from *K. variicola* (accession no. CP079802.1). Above the schematic depiction of genes is a G+C content plot and mutational response index plot. (**b**) Comparison of the ONovel32 cluster with the analogous cluster in *K. variicola* strain EMBL-1.

None of the ONovel32 clade isolates carried α-haemolysin or pyelonephritis-associated pili, which were present in ST131 and some of the other lineages. However, some VAGs were predominant among or restricted to ONovel32 strains (Fig. 4a, b). These include the *esp4L* type III secretion effector. We also searched independently for type III secretion systems and identified a cluster 97.5 % identical to *E. coli* type III secretion system 2, previously associated with virulence in septicaemic *E. coli* [47], in all *esp4L*-positive and one *esp4L*-negative Onovel32 isolate (GI8584164). *esp4L* was more common among these isolates than other phylogroup A strains, as well as multiple genes encoding capsular modification enzymes. All the ONovel32 strains and one associated ONT strain carried a *ugd* gene. The only other isolates in this collection with this gene were three isolates belonging to the globally disseminated high risk clone ST648 [48], known to possess biofilm-associated features that enhance pathogen emergence and persistence in both the human body and the environment. The *ugd* gene is associated with hypermucoviscosity and invasive virulence and the ONovel32 allele is 96 % identical to that from *K. pneumoniae* NTUH-K2044, a hypervirulent *K. pneumoniae* isolate [49], and *Klebsiella variicola* (accession no. CP079802.1) capsular cluster *ugd* genes. Six of the ONovel:32 strains (but not the ONT:H10 strain in the same clade) carried *cpsACP*, a chromosomally borne gene, which is predicted to encode a phosphatidic acid phosphatase (PAP2 Pfam 01569) family gene. PAP2 phosphatases replace phosphate groups on lipid A with amine groups, resulting in a positively charged lipid A that confers resistance to cationic peptides [50]. PAP2 phosphatases have been known to be transmitted horizontally solitarily or as part of capsular clusters [51]. A blast search revealed that the ONovel32 PAP2 allele is 99.6 % identical to endogenous PAP2 genes from *K. variicola* (accession no. CP079802.1). As shown in Fig. 5, depicting the region for ONovel32 ST1284 strain OAU-VOA-056, *cpsACP* is located within a capsular gene cluster identical to a *K. variicola* cluster and flanked by a 5´ IS*3* transposase and a 3´ IS*1* protein InsB-encoding gene. At the opposite end of the cluster is the *ugd* gene. The cluster shows G+C content and other base-pattern signatures that depart from the *E. coli* flanking sequence (Fig. 5a, b).

To determine whether the cluster is indeed novel, we genoserotyped the strains using two alternatives to srst2. As shown in Table S1, every non-ONovel32 strain received the same O-type designation from all three tools (including ONovel14 and ONovel27, which were returned as untypeable by the other two tools). However, ECTyper typed the srst2-designated ONovel32 isolates as O89 strains and CGE SerotypeFinder called them as O101 strains. O89- and O101-confering clusters are remarkably similar to one another [52]. Therefore, we retrieved the ONovel32, O89 and O101 antigen gene sequences from the EcOH, ECTyper and SerotypeFinder databases, respectively, and generated a maximum-likelihood phylogenetic tree from the muscle-generated alignment using mega x. The results show that the nearest neighbours to ONovel32 clusters defined by srst2 are O101 clusters from ECTyper (Fig. S4). However, the O89- and O101-conferring clusters do not resolve into separate clades and, therefore, the serotype of strains bearing either cluster cannot be predicted with complete confidence. Interestingly, a systematic literature search revealed that an ST167 ONovel32 strain (strain Sanji, referred to as O89-like, O89b) has previously caused a colibacillosis outbreak in pheasants in China [53]. Pairwise alignment of that strain’s O-antigen cluster (GenBank accession no. CP011061.1) with that of ONovel32 strain G18484079 revealed that they are virtually identical and point to the likelihood that these ExPEC strains, like many other ExPEC lineages, have avian pathogenic potential. Zeng *et al*. identified a number of other ST167, ST617, ST10, ST1284 and other strains carrying this O-antigen cluster [53]. Interestingly, according to Zeng *et al*., strain Sanji genoserotyped as O89b but immunoserotyped as O6. O101 is known to be expressed, O89 has been previously reported as containing a complete but unexpressed O-antigen cluster, and Sanji’s O89b remains unevaluated phenotypically [54]. Observing that ONovel:32/O101/O89b cluster isolates from our own study, some of which were originally misclassified as *Klebsiella *, showed mucoidity upon plate culture, we sought to determine whether these strains had distinctive colonization-associated capacities. The median biofilm *A*_570_ at 6 h was 0.178 for phylogroup A strains lacking *cpsACP* and 0.216 for those with the gene (Fig. 2), but these differences were not significant. All the isolates were also negative in the string test for hypermucoviscosity in *Klebsiella* [55].

### Resistance profiles and concordance with predicted AMR

**Fig. 6.**
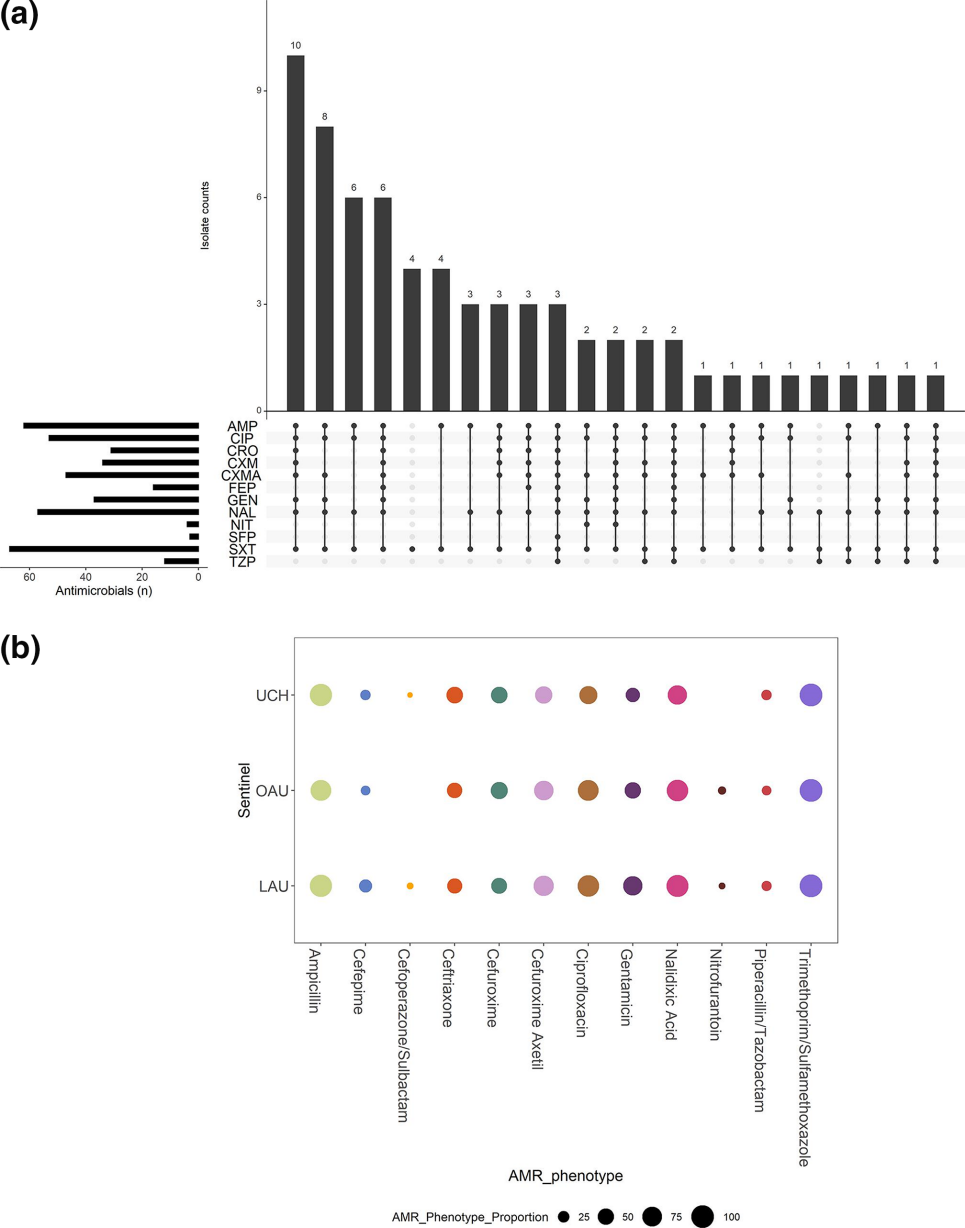
Resistance profile of ExPEC isolates. The main bar chart demonstrates the number of ExPEC isolates with each combination of resistance to tested antibiotics, and is ordered in descending order by the frequency of resistance profiles observed among 67 ExPEC isolates. The side bar chart demonstrates the number of isolates that are resistant to each of the named antibiotics. The dots and lines between dots at the base of the main bar chart (and the right side of the side bar chart) show the co-resistance status of the ExPEC isolates. All isolates were susceptible to amikacin, meropenem, imipenem and ertapenem. AMP, Ampicillin; CIP, ciprofloxacin; CRO, ceftriaxone; CXM, cefuroxime; CXMA, cefuroxime axetil; FEP, cefepime; GEN, gentamicin; NAL. nalidixic acid; NIT, nitrofurantoin; SFP, cefoperazone/sulbactam; SXT, trimethoprim/sulfamethoxazole; TZP, piperacillin/tazobactam. (b) AMR phenotypes of ExPEC Isolates, stratified by sentinel site [LAU (*n*=28), OAU (*n*=22), UCH (*n*=21)]. The size of the coloured circles represents the proportion of isolates recovered from each sentinel site that demonstrated resistance to the tested antibiotics.

**Fig. 7.**
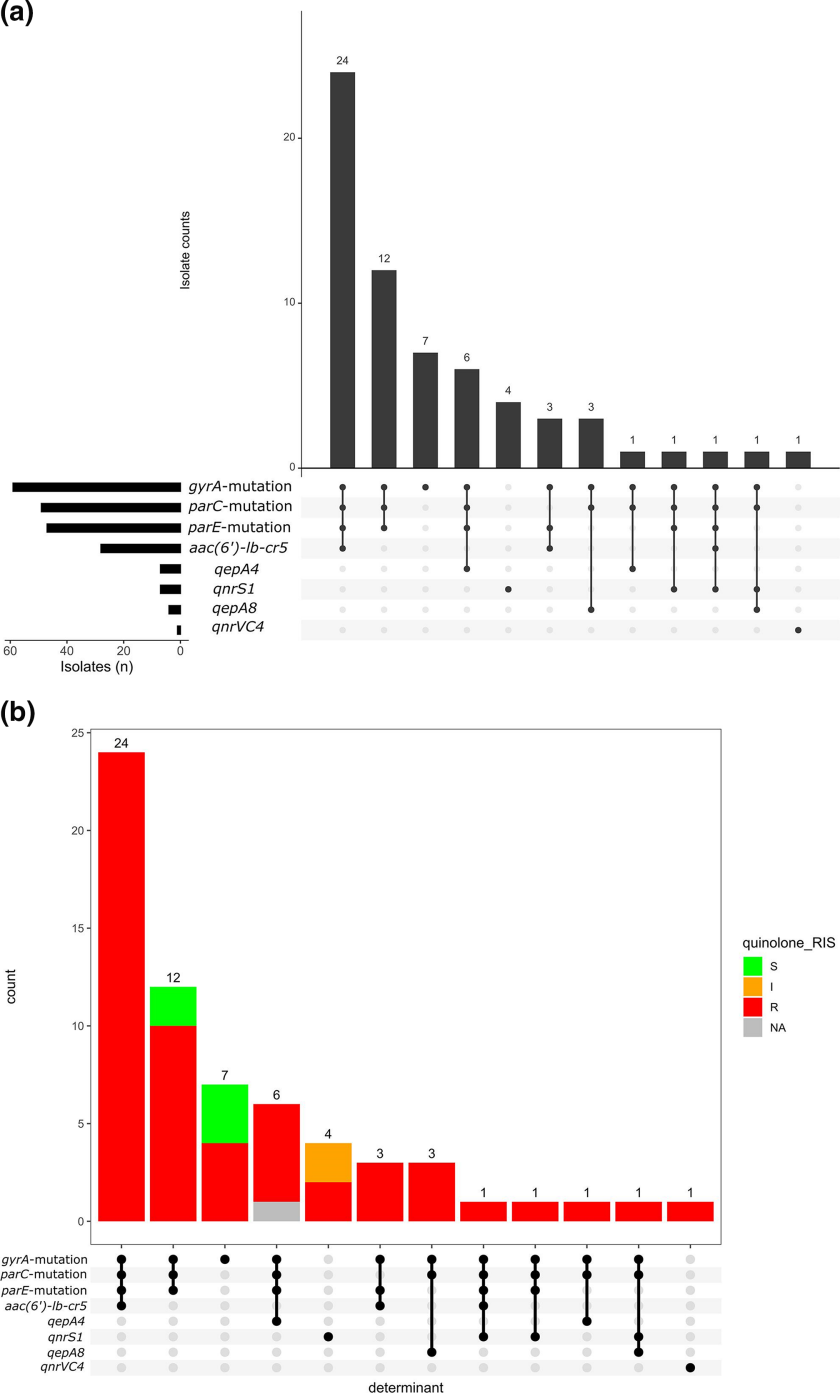
Quinolone-resistance gene combinations in ExPEC isolates. The *upset* plots (a, b) show the number of ExPEC isolates carrying each combination of genes conferring resistance to the quinolones, and (b) is coloured by the proportion of observed phenotypic antimicrobial susceptibility, and is ordered in descending order by the frequency of resistance gene profiles observed. The side bar chart in (a) demonstrates the number of isolates that carry each of the resistance genes. The dots and lines between dots at the base of the main bar chart (and the right side of the side bar chart) show the co-resistance gene profile of the ExPEC isolates. NA, phenotypic antimicrobial-susceptibility data for the isolate is absent.

Susceptibility testing of 67 out of the 68 isolates showed that, of the 16 antibiotics tested, resistance to trimethoprim/sulfamethoxazole (*n*=67; 100 %), ampicillin (*n*=62; 92.5 %), nalidixic acid (*n*=57; 85.1 %) and ciprofloxacin (*n*=53; 79.1 %) was commonly observed (Fig. 6a, b, Table S2). However, resistance to cefoperazone/sulbactam (*n*=3; 4.5 %) was less common. All isolates were susceptible to amikacin, ertapenem and meropenem (Table S3, Fig. S2a and S2b). One *bla_CTX-M-15_* -positive isolate from LAU (ST11025, B1, O51:H12) was categorized as showing intermediate to imipenem but did not carry any carbapenemase-producing gene. Resistance profiles were remarkably similar among the three sentinels (Fig. 6b). Resistance to trimethoprim/sulfamethoxazole among isolates belonging to all 33 STs could be explained by the possession of *dfrA* (*n*=54/67) and *dfrB* (*n*=2/67) genes. Ciprofloxacin resistance (23 STs) was largely attributable to mutation in the quinolone resistance determining regions (QRDRs) of *gyrA* (D87N, S83L), *parC* (E84A, E84G, E84K, S57T, S80I) and/or *parE* (E460D, I355T, I529L, I529L, I464F, L416F, L445H, S458A, S458T), with or without the presence of plasmid quinolone-resistance genes [*qnrS*, *qnrVC4*, *qepA*, *aac-(6’)-Ib-cr*]. About 45 % (*n*=24/53) of isolates phenotypically resistant to ciprofloxacin carried a combination of *aac(6’)-lb-cr5* gene and mutation in the QRDRs (*gyrA*, *parC* and *parE*) (Fig. 7a, b). The most common quinolone-resistance gene profile observed is the ‘gyrA_D87N, gyrA_S83L, parC_S80I, parE_S458A’, as observed in a quarter (*n*=14/53) of isolates showing phenotypic resistance to ciprofloxacin.

Ampicillin resistance among isolates belonging to 29 STs (*n*=62) could be explained by the carriage of a range of β-lactamase genes including *bla_TEM-1_* (36/62 of isolates), *bla_TEM-40_* (3 isolates), *bla_TEM-84_* (2 isolates), *bla_TEM-135_* (2 isolates) or *bla_OXA-2_* (co-occurring with *bla_TEM-90_*; 1 isolate). Extended-spectrum β-lactam resistance likely resulted from *bla_CTX-M-15_* (33 isolates), *bla_CTX-M-27_* (1 isolate), *bla_VEB-1_* (1 isolate) and/or *bla_CMY-42_* (2 isolates) (Fig. S2a, b) co-occurring largely with the plasmids Inc types FIB_AP001918, FIA, IncFI, IncQ1, IncFII_p and the col plasmid Col156.

Of the five drug classes tested, the highest concordance between phenotypic resistance and predicted AMR was observed for trimethoprim (100 % concordance, TP=67/67, sensitivity=100 %, specificity=not available) and the lowest concordance for drugs within the aminoglycoside class (concordance=55.22 %, TP=37/67, sensitivity=100 %, specificity=0 %) (Table S3a). The genes predicted to confer resistance to antibiotics belonging to the aforementioned drug classes can be found in Table S3(b).

### Multidrug resistance (MDR)

We observed a total of 23 resistance profiles (Table S1) with the profiles in many cases associated with specific STs (Table S1). Fifty-nine (88.06 %) of the isolates were resistant to at least one agent within at least three classes of antibiotics, fitting the MDR definition of the international AMR community [56] (Table S1). Isolates belonging to the most frequent STs form the bulk of bloodstream isolates carrying extended-spectrum β-lactamase (ESBL) genes or mutations in the QRDRs (*gyrA*, *parC*, *parE*), and the plasmid-mediated quinolone-resistance gene *aac(6’)-lb-cr*, as well as genes conferring resistance to trimethoprim. Carbapenemase genes were conspicuously absent, in agreement with the AMR phenotype. Almost all isolates carrying ESBL genes (*n*=32/34) are multidrug resistant. About 30 % of these showed the most frequent resistance profile (RP-1) (Fig. S3). Furthermore, multidrug-resistant bloodstream isolates do not carry the same resistance determinants and plasmid replicons, apart from *gyrA/parC/parE* mutations (Fig. S3).

Resistance-gene profiles differed significantly between the two ST131 clades (Fig. 3c). Unlike ST131 lineage 1 isolates, ST131 lineage 2 isolates did not carry any β-lactamase gene besides *ampC*. All but one of ST131 lineage 2 isolates carried the ESBL gene *bla_CTX-M-15_* (Fig. 3c). Within the ST131 lineage 1, two isolates did not carry genes conferring resistance/reduced susceptibility to the aminoglycosides, phenicols, macrolides and quaternary ammonium compounds. Furthermore, the absence of the Col156, IncB_O_K_Z and IncFI plasmids in these two isolates seem to have been compensated by the possession of IncFIA, IncFII_p and IncI1 plasmids, noted to have been absent in the other three ST131 isolates within the ST131 lineage 1.

All the ONovel32 strains (but not the co-clustering ONT strain) carried one ESBL gene, *bla_CTX-M-15_*, and these strains also carried IncF plasmids, common among phylogroup A strains (Table S1). Every one of them carried the most common four resistance-conferring mutations in the QRDRs (gyrA_D87N, gyrA_S83L, parC_S80I, parE_S458A) and seven, including the ONT strain, carried *qepA4*. Four ONovel32 isolates additionally carried *aac-(6’)-Ib-cr*, conferring aminoglycoside and ciprofloxacin resistance, as well as horizontally transmitted genes conferring resistance to trimethoprim, chloramphenicol and tetracyclines were common (https://microreact.org/project/hmj3KwxS1dmmFPCKFx6qeA-invasive-escherichia-coli-sw-nigeria-2016-2018, Table S1).

Common to both the ST131 and the ONovel:32 clades is the high prevalence of *qacEdelta1* (*n*=9 and 8, respectively) conferring resistance to quaternary ammonium compounds, commonly used for disinfecting hospital surfaces and associated with class 1 integrons. Altogether, these clades comprised 17 (42.50 %) of 40 *E. coli* isolates carrying this gene.

## Discussion

This study characterized 68 bloodstream *E. coli* isolates as an important first step in understanding their epidemiology within South-West Nigeria. In this small collection, we identified multiple clones of pandemic importance, and found two predominant clades. One of these, comprising two ST131 lineages, is globally disseminated and this study illustrates its importance in Nigerian health facilities. The second predominant clade does not feature in present discourse on international ExPEC clones, and represents strains belonging to ST10, ST167 and related STs, which predominantly encode O-antigen genes recently reported from avian pathogenic *E. coli* in China. In addition to these prominent clades, we identified strains belonging to major pandemic ExPEC lineages, including ST12, ST73 and ST648, and their single locus variants. ST69, ST95 and ST405 lineages were not detected but our sample is not very large and, therefore, our findings are insufficient to rule them epidemiologically insignificant in our setting. In the UK and some high-income countries, ST69, ST73, ST95 and ST131 lineages have attained sufficient population stability independent of the acquisition of AMR genes [6, 57]. We additionally identified in the collection five ST90/410 strains, all from a single facility. Two ST90 isolates are identical, differ from a third by only 11 SNPs and likely represent an outbreak. Our findings add to information that is chronicling ExPEC lineages of importance within Nigeria [14–16, 58, 59], other parts of Africa [16, 60] and other LMICs [16, 61].

Among ST131, we found both globally disseminated lineages within our isolate collection. The majority of haemolytic and *pap* gene-bearing phylogroup B2 strains belonged to this ST. Biofilm formation among these strains was common and associated with the *kpsD* gene, a known contributor to biofilm formation [62]. The ST10, ST167 and related strains that comprised the ONovel32/O101/O89b clade were distinguished by the presence of one of two variations of a capsular island that has been well described in *Klebsiella* with biofilm formation among them being more pronounced among strains carrying the *cpsACP*-containing portion of the island. While the virulence of ST131 has been well described, features of this clade that cause it to predominate remain unknown, and further studies are required to understand its pathogenicity and selective success in our setting.

O101 and O89 gene clusters are highly similar and not resolvable phylogenetically, and include the ONovel32 cluster found in this study. While the O89 cluster is reputed to be intact but not expressed, O89b, which is essentially identical to the cluster found in this study, has been found in the genome of O-antigen-expressing avian pathogenic *E. coli* (APEC)strains [53]. The O89/O101 antigen gene cluster (as well as O8 and O9) is thought to have been transferred from *Klebsiella* to *E. coli* via horizontal gene transfer [63], and requires better characterization in the *E. coli* background in order to produce a correct and congruent output from existing *in silico* serotyping tools. The O101:H10 *fimH54* clone has been reported to carry ESBL genes and the *gyrA/parC* mutations [64], which confe resistance to last linantibiotics commonly used in our setting. O89b strain Sanji is extensively drug resistant and the ONovel32/O89b/O101 strains in this study had multiple AMR gene profiles.

High levels of phenotypic resistance to antibiotics within the antifolate (trimethoprim, co-trimoxazole), quinolone (nalidixic acid), fluoroquinolone (ciprofloxacin), cephalosporin (cefuroxime axetil) and the aminoglycoside (gentamicin) classes observed among bloodstream isolates in this study corroborates reports from previous studies [12, 58, 59], and poses a serious concern for clinical therapeutics. *In silico* data confirm the abundance of genes conferring resistance, with many isolates carrying more than one gene conferring resistance to a specific antimicrobial or class. Before the turn of the millennium, resistance to many of the aforementioned antimicrobials was rare in Africa. However, steady increase in the availability and use of these agents in the empiric treatment of ExPEC-related infections has inevitably selected for AMR. The increased rate of fluoroquinolone resistance in diarrhoeagenic *E. coli* and other *Enterobacterales *, for instance, coincided with increased fluoroquinolone use in Nigeria [65] and other parts of Africa [7, 66]. While findings from a study conducted in Nigeria more than a decade ago [13] concluded that nalidixic acid was still an effective antimicrobial, we observed in this study that resistance to nalidixic acid is now common. Similarly, cephalosporin resistance emerged and expanded much more in Africa than in other parts of the world, as these agents became the drug of choice for treating multidrug-resistant pathogens [66].

Fluoroquinolone resistance has also been previously associated with the presence of ESBL genes, because ESBL genes are often borne on transferable large plasmids that co-host some of the PMQR genes [67]. We observed the co-carriage of ESBL and fluoroquinolone-resistance genes in more than a quarter of the ExPEC isolates, and particularly in over-represented lineages.

We find that both predominant lineages we have highlighted in this report show multiple resistance. This has important implications for patients with life-threatening bloodstream infections and provides a plausible explanation for their evolutionary success in our setting. Resistance to antimicrobials used intensively in Nigeria (trimethoprim, aminopenicillins and ciprofloxacin) was rife, and resistance to the agents most frequently used empirically when blood stream infections are suspected – second- and third-generation cephalosporins and aminoglycosides – was also worryingly common. While none of these clades showed carbapenem or colistin resistance, these reserve antimicrobial classes are out of the reach of most patients attending the three hospitals from which the strains were obtained.

Next-generation sequencing has emerged as a promising complement to clinical bacteriology as it provides answers to medical conundrums, as well as a more robust picture of the epidemiology of infectious diseases. It can also reveal, as in this case, circulation of hitherto unrecognized clones of concern. Although its integration into clinical diagnosis and patient care gaining ground in many parts of the world, its adoption in LMICs (and the rest of the globe) is still hindered by lack of infrastructure, cost of implementing WGS, limited bioinformatics expertise and as yet mildly inaccurate prediction of AMR [68, 69]. In our study, we observed perfect concordance (100 %) with phenotypic AST data for trimethoprim, but not for the cephalosporins, the quinolones or the aminoglycosides. Therefore, this shows that further understanding of resistance mechanisms and routine AMR database update are needed to enhance the feasibility of gradual and sustained integration of WGS into routine diagnosis.

Another advantage of next-generation sequencing is its potential in rapid detection of outbreaks either retrospectively as is more common, or potentially (but not yet achievable in our own system) in real-time. We report a likely retrospective ST90 outbreak in LAU, which was not detected using traditional diagnostic methods in the sentinel laboratories. ST90 strains have variously been highlighted for their zoonotic potential and association with device-related hospital outbreaks [70–72], and their epidemiology in our setting remains to be understood; ST90, and related ST410, were only seen at one facility and the representation of ST90 at this facility may have been amplified by an outbreak. The adoption of genomic surveillance in diagnostic laboratories within Nigeria will ensure that outbreaks of this and other clones can be detected in real time, while definite tracking and containment of the spread of such clones will be achieved before lives are lost.

This study has a few limitations. Blood culture is infrequently performed in Nigerian hospitals and, until recently, most isolates were not archived. Therefore, these isolates represent but a fraction of the ExPEC likely to have infected patients in the three hospitals and may not be representative. Our short-read data makes it impossible to accurately determine whether the resistance genes are located on the bacterial chromosome or plasmids (or other mobile genetic elements). In future, we will incorporate long-read sequencing into our prospective surveillance efforts in order to correctly identify plasmid-borne AMR genes.

In conclusion, this study has provided hospital-specific information on the population structure of ExPEC lineages needed to track pandemic lineages and guide infection disease control practices in line with Nigeria’s national action plan on AMR.

## Supplementary Data

Supplementary material 1

Supplementary material 2
