# Supplementary material for: An ST131 clade and a phylogroup A clade bearing an O101-like O-antigen cluster predominate among bloodstream Escherichia coli isolates from South-West Nigeria hospitals
Source: Microb Genom. 2022 Dec 16;8(12):mgen000863. doi: 10.1099/mgen.0.000863 (PMC9837563; doi:10.1099/mgen.0.000863)
Supplement: Supplementary material 1 [file mgen-8-863-s001.pdf]

Title. An ST131 clade and a Phylogroup A clade bearing O101-like O-antigen cluster predominate among bloodstream *Escherichia coli* isolates from southwest Nigeria hospitals.

Ayorinde O. Afolayan, Aaron O. Aboderin, Anderson O. Oaikhena, Erkison Ewomazino Odih, Veronica O. Ogunleye, Adeyemi T. Adeyemo, Abolaji T. Adeyemo, Oyeniyi S. Bejide, Anthony Underwood, Silvia Argimón, Monica Abrudan, Abiodun Egwuenu, Chikwe Ihekweazu, David M. Aanensen and Iruka N. Okeke

Supplementary data files

Table S1 is a spreadsheet containing epidemiological data, antimicrobial susceptibility data, and *in silico* data (AMR, virulence, plasmids, serotype, fimtype, phylogroup)

Table S2: Summary of Antibiotic Susceptibility Test data showing the number of *E. coli* isolates that were resistant to the named antibiotics.

Drug class	Drug	Resistance % (resistance (n)/species (n))
Aminoglycosides	Gentamicin (GEN)	55.20% (37/67)
Beta-lactams/penicillins	Ampicillin (AMP)	92.50% (62/67)
	Piperacillin/Tazobactam (TZP)	17.90% (12/67)
Cephalosporins (2nd generation)	Cefuroxime (CXM)	50.70% (34/67)
	Cefuroxime Axetil (CXMA)	70.10% (47/67)
Cephalosporins (3rd generation)	Cefoperazone-Sulbactam (SFP)	4.50% (3/67)
	Ceftriaxone (CRO)	46.30% (31/67)
Cephalosporins (4th generation)	Cefepime (FEP)	23.90% (16/67)
Other antibacterials	Nitrofurantoin (NIT)	6.00% (4/67)
Quinolones	Ciprofloxacin (CIP)	79.10% (53/67)
	Nalidixic acid (NAL)	85.10% (57/67)
Trimethoprims	Trimethoprim/sulfamethoxazole (SXT)	100.0% (67/67)

Table S3a: Degree of Agreement between phenotypic antimicrobial susceptibility tests and genotypic predicted antimicrobial resistance

drug	Tests (N)	concordance (%)	TP	FP	TN	FN	specificity	Specificity lower limits	Specificity upper limits	sensitivity	Sensitivity lower limits	Sensitivity upper limits
Aminoglycosides	67	55.22	37	30	0	0	0	0	0.115703308	1	0.905109413	1
Beta-lactams	67	71.64	45	1	3	18	0.75	0.19412045	0.993690537	0.714285714	0.586538974	0.821070793
Cephalosporins	67	68.66	46	20	0	1	0	0	0.168433471	0.978723404	0.887062283	0.999461468
Quinolones	67	86.57	56	7	2	2	0.222222222	0.028144973	0.600093574	0.965517241	0.880922689	0.995796353
Trimethoprim	67	100	67	0	0	0	NA	0	1	1	0.946430346	1

Table S3b: List of genes predicted to confer resistance to antibiotics belonging to the drug classes in Table S3a

**Drug Classes**	**AMR genes**
Aminoglycosides	*aac(6')-Ie/aph(2'')-Ia*, *aac(6')-Ib-cr5*, *catB8/aac(6')-Ib'*, *aac(6')-30/aac(6')-Ib'*, *aac(3)-IIe*, *aac(3)-IId*, *aac(6')-Ib4*, *ant(2'')-Ia*, *aph(3')-Ia*, *aadA2*, *aph(3'')-Ib*, *aph(6)-Id*, *aadA5*, *aadA13*, *aadA1*, *aadA8*
Beta-lactams	$bla_{\text{TEM-1}}$, $bla_{\text{OXA-2}}$, $bla_{\text{TEM-90}}$, $bla_{\text{TEM-57}}$, $bla_{\text{TEM-40}}$, $bla_{\text{TEM-84}}$, *blaZ*, *blaR1*, $bla_{\text{TEM-135}}$
Cephalosporins	$bla_{\text{CTX-M-15}}$, $bla_{\text{EC-15}}$, $bla_{\text{EC-5}}$, $bla_{\text{OXA-1}}$, $bla_{\text{EC-16}}$, $bla_{\text{EC-18}}$, $bla_{\text{CTX-M-27}}$, $bla_{\text{EC-8}}$, $bla_{\text{CMY-2}}$, $bla_{\text{EC-19}}$, $bla_{\text{CMY-42}}$, $bla_{\text{TEM-187}}$, $bla_{\text{VEB-1}}$
Quinolones	*gyrA*, *parC*, and *parE* mutations, *qepA4*, *qnrS1*, *qepA8*, *qnrVC4*
Trimethoprim	*dfrA12*, *dfrA17*, *dfrA7*, *dfrA14*, *dfrA29*, *dfrF*, *dfrB4*, *dfrA5*, *dfrA1*

Supplementary Table 4: List of 159 Virulence-associated Genes (VAGs), Reference, and Accession Numbers

VAGs	Isolate No	Accession Numbers	Reference
entB	67	WP_001007140	http://www.mgc.ac.cn/cgi-bin/VFs/gene.cgi?GeneID=VFG000933
entC	67	WP_001336900	http://www.mgc.ac.cn/cgi-bin/VFs/gene.cgi?GeneID=VFG000931
fepA	67	WP_001034892	http://www.mgc.ac.cn/cgi-bin/VFs/gene.cgi?GeneID=VFG000923
fepB_1	67	WP_001234311	http://www.mgc.ac.cn/cgi-bin/VFs/gene.cgi?GeneID=VFG000924
fepC	67	WP_000140634	http://www.mgc.ac.cn/cgi-bin/VFs/gene.cgi?GeneID=VFG000925
fepG	67	WP_000640938	http://www.mgc.ac.cn/cgi-bin/VFs/gene.cgi?GeneID=VFG000928
fepD	66	WP_001443194	http://www.mgc.ac.cn/cgi-bin/VFs/gene.cgi?GeneID=VFG000926
entE_1	65	WP_000026784	http://www.mgc.ac.cn/cgi-bin/VFs/gene.cgi?GeneID=VFG000932
entS	65	WP_001041793	http://www.mgc.ac.cn/cgi-bin/VFs/gene.cgi?GeneID=VFG044165
fes_1	65	WP_000125846	http://www.mgc.ac.cn/cgi-bin/VFs/gene.cgi?GeneID=VFG044159
ompA_1	65	AAF37887	http://www.mgc.ac.cn/cgi-bin/VFs/gene.cgi?GeneID=VFG001443
yagV_ecpE	60	WP_001301550	http://www.mgc.ac.cn/cgi-bin/VFs/gene.cgi?GeneID=VFG002417
yagW_ecpD	60	WP_001265657	http://www.mgc.ac.cn/cgi-bin/VFs/gene.cgi?GeneID=VFG002416
yagZ_ecpA	60	WP_000730972	http://www.mgc.ac.cn/cgi-bin/VFs/gene.cgi?GeneID=VFG002414
ykgK_ecpR	60	WP_000389022	http://www.mgc.ac.cn/cgi-bin/VFs/gene.cgi?GeneID=VFG002413
yagY_ecpB	59	WP_000716386	http://www.mgc.ac.cn/cgi-bin/VFs/gene.cgi?GeneID=VFG002412
yagX_ecpC	58	WP_001131063	http://www.mgc.ac.cn/cgi-bin/VFs/gene.cgi?GeneID=VFG002415
gspL	55	WP_000097229	http://www.mgc.ac.cn/cgi-bin/VFs/gene.cgi?GeneID=VFG002055
fimF_1	54	WP_001244821	http://www.mgc.ac.cn/cgi-bin/VFs/gene.cgi?GeneID=VFG000877
fimG	54	WP_000872015	http://www.mgc.ac.cn/cgi-bin/VFs/gene.cgi?GeneID=VFG000878
fimH_1	54	WP_000832235	http://www.mgc.ac.cn/cgi-bin/VFs/gene.cgi?GeneID=VFG000879
gspK	54	WP_000633233	http://www.mgc.ac.cn/cgi-bin/VFs/gene.cgi?GeneID=VFG002054
fimC	53	WP_000066579	http://www.mgc.ac.cn/cgi-bin/VFs/gene.cgi?GeneID=VFG000875
fimE_2	53	WP_000044711	http://www.mgc.ac.cn/cgi-bin/VFs/gene.cgi?GeneID=VFG000872
fimD_3	52	WP_000120946	http://www.mgc.ac.cn/cgi-bin/VFs/gene.cgi?GeneID=VFG000876
fimI_1	52	WP_000824100	http://www.mgc.ac.cn/cgi-bin/VFs/gene.cgi?GeneID=VFG000874
gspC	50	WP_000135089	http://www.mgc.ac.cn/cgi-bin/VFs/gene.cgi?GeneID=VFG002046
gspF	50	WP_001173470	http://www.mgc.ac.cn/cgi-bin/VFs/gene.cgi?GeneID=VFG002049
gspD	49	WP_000498818	http://www.mgc.ac.cn/cgi-bin/VFs/gene.cgi?GeneID=VFG002047
gspG	49	WP_001087302	http://www.mgc.ac.cn/cgi-bin/VFs/gene.cgi?GeneID=VFG002050
gspM	49		http://www.mgc.ac.cn/cgi-bin/VFs/gene.cgi?GeneID=VFG002056
gspI	48	WP_000820136	http://www.mgc.ac.cn/cgi-bin/VFs/gene.cgi?GeneID=VFG002052
fimB_2	45	WP_000790574	http://www.mgc.ac.cn/cgi-bin/VFs/gene.cgi?GeneID=VFG000871
espX5	43	WP_001270084	http://www.mgc.ac.cn/cgi-bin/VFs/gene.cgi?GeneID=VFG034873
gspE	43	WP_000249362	http://www.mgc.ac.cn/cgi-bin/VFs/gene.cgi?GeneID=VFG002048
gspH	43	WP_001115126	http://www.mgc.ac.cn/cgi-bin/VFs/gene.cgi?GeneID=VFG002051
entD	40	WP_001375021	http://www.mgc.ac.cn/cgi-bin/VFs/gene.cgi?GeneID=VFG000929
fyuA	39	WP_000784549	http://www.mgc.ac.cn/cgi-bin/VFs/gene.cgi?GeneID=VFG012567
fdeC	38	WP_000092542	http://www.mgc.ac.cn/cgi-bin/VFs/gene.cgi?GeneID=VFG045349
ybtA	38	WP_000970688	http://www.mgc.ac.cn/cgi-bin/VFs/gene.cgi?GeneID=VFG012543
ybtE	38		http://www.mgc.ac.cn/cgi-bin/VFs/gene.cgi?GeneID=VFG012563

ybtP	38	WP_001327262	http://www.mgc.ac.cn/cgi-bin/VFs/gene.cgi?GeneID=VFG012539
ybtQ	38	WP_001327263	http://www.mgc.ac.cn/cgi-bin/VFs/gene.cgi?GeneID=VFG012535
ybtS	38	WP_001327263	http://www.mgc.ac.cn/cgi-bin/VFs/gene.cgi?GeneID=VFG012535
ybtT	38	WP_000194283	http://www.mgc.ac.cn/cgi-bin/VFs/gene.cgi?GeneID=VFG012559
ybtU	38	WP_000194283	http://www.mgc.ac.cn/cgi-bin/VFs/gene.cgi?GeneID=VFG012559
ybtX	38	WP_001286281	http://www.mgc.ac.cn/cgi-bin/VFs/gene.cgi?GeneID=VFG012531
irp1	36	WP_000369487	http://www.mgc.ac.cn/cgi-bin/VFs/gene.cgi?GeneID=VFG034120
irp2	33	WP_000623043	http://www.mgc.ac.cn/cgi-bin/VFs/gene.cgi?GeneID=VFG034104
espX1	29	WP_000129338	http://www.mgc.ac.cn/cgi-bin/VFs/gene.cgi?GeneID=VFG034849
iucC	29	WP_001015709	http://www.mgc.ac.cn/cgi-bin/VFs/gene.cgi?GeneID=VFG000938
iucB	28	WP_001287497	http://www.mgc.ac.cn/cgi-bin/VFs/gene.cgi?GeneID=VFG000939
chuA	27	WP_000089583	http://www.mgc.ac.cn/cgi-bin/VFs/gene.cgi?GeneID=VFG000917
chuS	27	WP_001017192	http://www.mgc.ac.cn/cgi-bin/VFs/gene.cgi?GeneID=VFG000916
chuT	27	WP_001312167	http://www.mgc.ac.cn/cgi-bin/VFs/gene.cgi?GeneID=VFG000918
chuU	27	WP_000910402	http://www.mgc.ac.cn/cgi-bin/VFs/gene.cgi?GeneID=VFG000922
chuV	27	WP_000622316	http://www.mgc.ac.cn/cgi-bin/VFs/gene.cgi?GeneID=VFG044172
chuW	27	WP_001095619	http://www.mgc.ac.cn/cgi-bin/VFs/gene.cgi?GeneID=VFG000919
chuX	27	WP_000020034	http://www.mgc.ac.cn/cgi-bin/VFs/gene.cgi?GeneID=VFG000920
chuY	27	WP_000189356	http://www.mgc.ac.cn/cgi-bin/VFs/gene.cgi?GeneID=VFG000921
papX	27	WP_001298062	http://www.mgc.ac.cn/cgi-bin/VFs/gene.cgi?GeneID=VFG001713
iucA	26	WP_001299620	http://www.mgc.ac.cn/cgi-bin/VFs/gene.cgi?GeneID=VFG000940
iutA_1	26	WP_016265865	http://www.mgc.ac.cn/cgi-bin/VFs/gene.cgi?GeneID=VFG000936
kpsD	25	WP_001331697	http://www.mgc.ac.cn/cgi-bin/VFs/gene.cgi?GeneID=VFG050159
papI	25	WP_000006206	http://www.mgc.ac.cn/cgi-bin/VFs/gene.cgi?GeneID=VFG000880
gspJ	23	WP_000082782	http://www.mgc.ac.cn/cgi-bin/VFs/gene.cgi?GeneID=VFG040938
papC	23	WP_000654937	http://www.mgc.ac.cn/cgi-bin/VFs/gene.cgi?GeneID=VFG000895
papD	23	WP_000265729	http://www.mgc.ac.cn/cgi-bin/VFs/gene.cgi?GeneID=VFG000896
papF	23	WP_000113351	http://www.mgc.ac.cn/cgi-bin/VFs/gene.cgi?GeneID=VFG000889
papJ	23	WP_000261297	http://www.mgc.ac.cn/cgi-bin/VFs/gene.cgi?GeneID=VFG000886
papK	23	WP_000597713	http://www.mgc.ac.cn/cgi-bin/VFs/gene.cgi?GeneID=VFG000887
papG	21	WP_000758683	http://www.mgc.ac.cn/cgi-bin/VFs/gene.cgi?GeneID=VFG000890
espL1	18	WP_000081129	http://www.mgc.ac.cn/cgi-bin/VFs/gene.cgi?GeneID=VFG034754
sat	17	WP_001034089	http://www.mgc.ac.cn/cgi-bin/VFs/gene.cgi?GeneID=VFG000902
cluster_3	15		
gnd	15	WP_014907233	http://www.mgc.ac.cn/cgi-bin/VFs/gene.cgi?GeneID=VFG048830
kpsM_1	15	WP_000124301	http://www.mgc.ac.cn/cgi-bin/VFs/gene.cgi?GeneID=VFG050169
senB	15	WP_001020413	http://www.mgc.ac.cn/cgi-bin/VFs/gene.cgi?GeneID=VFG036085
espL4	14	WP_000627910	http://www.mgc.ac.cn/cgi-bin/VFs/gene.cgi?GeneID=VFG034772
ugd	14	WP_004175261	http://www.mgc.ac.cn/cgi-bin/VFs/gene.cgi?GeneID=VFG048797
fimA	13	WP_000695571	http://www.mgc.ac.cn/cgi-bin/VFs/gene.cgi?GeneID=VFG000873
entF	11	WP_000077767	http://www.mgc.ac.cn/cgi-bin/VFs/gene.cgi?GeneID=VFG000930
espX4	11	WP_000900547	http://www.mgc.ac.cn/cgi-bin/VFs/gene.cgi?GeneID=VFG034866
papE	11	WP_000723799	http://www.mgc.ac.cn/cgi-bin/VFs/gene.cgi?GeneID=VFG000888
papH	11	WP_001239363	http://www.mgc.ac.cn/cgi-bin/VFs/gene.cgi?GeneID=VFG000894
iroD	10	WP_000933673	http://www.mgc.ac.cn/cgi-bin/VFs/gene.cgi?GeneID=VFG012505
iroE	10	WP_000271272	http://www.mgc.ac.cn/cgi-bin/VFs/gene.cgi?GeneID=VFG012501

manC_1	10	WP_003022279	http://www.mgc.ac.cn/cgi-bin/VFs/gene.cgi?GeneID=VFG047006
espR1	9	WP_000671689	http://www.mgc.ac.cn/cgi-bin/VFs/gene.cgi?GeneID=VFG034815
hlyA	8	WP_000217739	http://www.mgc.ac.cn/cgi-bin/VFs/gene.cgi?GeneID=VFG000840
hlyB	8	WP_000217739	http://www.mgc.ac.cn/cgi-bin/VFs/gene.cgi?GeneID=VFG000840
hlyD_1	8	WP_001213545	http://www.mgc.ac.cn/cgi-bin/VFs/gene.cgi?GeneID=VFG000843
iroB	8	WP_001221122	http://www.mgc.ac.cn/cgi-bin/VFs/gene.cgi?GeneID=VFG012513
iroC	8	WP_001111200	http://www.mgc.ac.cn/cgi-bin/VFs/gene.cgi?GeneID=VFG012509
iroN	8	WP_001222189	http://www.mgc.ac.cn/cgi-bin/VFs/gene.cgi?GeneID=VFG000935
galF	7	WP_001741945	http://www.mgc.ac.cn/cgi-bin/VFs/gene.cgi?GeneID=VFG048990
astA	6	AB042002.1	https://www.ncbi.nlm.nih.gov/protein/BAA94855
cpsACP	6	WP_014907219	https://www.ncbi.nlm.nih.gov/protein/WP_014907219.1?
vat	6	WP_001034006	http://www.mgc.ac.cn/cgi-bin/VFs/gene.cgi?GeneID=VFG000904
clbP	5	WP_002430641	http://www.mgc.ac.cn/cgi-bin/VFs/gene.cgi?GeneID=VFG049162
clbQ	5	WP_000065646	http://www.mgc.ac.cn/cgi-bin/VFs/gene.cgi?GeneID=VFG049163
clbS	5	WP_004175305	http://www.mgc.ac.cn/cgi-bin/VFs/gene.cgi?GeneID=VFG049164
cnf1	5	CAA50007	http://www.mgc.ac.cn/cgi-bin/VFs/gene.cgi?GeneID=VFG001447
espY2	5	WP_000868321	http://www.mgc.ac.cn/cgi-bin/VFs/gene.cgi?GeneID=VFG034901
manB_1	5	WP_004899416	https://www.ncbi.nlm.nih.gov/protein/WP_004899416.1?
tcpC	5	WP_000562556	http://www.mgc.ac.cn/cgi-bin/VFs/gene.cgi?GeneID=VFG045464
clbA	4	WP_001217110	http://www.mgc.ac.cn/cgi-bin/VFs/gene.cgi?GeneID=VFG049147
clbB	4	WP_041168941	http://www.mgc.ac.cn/cgi-bin/VFs/gene.cgi?GeneID=VFG049148
clbC	4	WP_041169023	http://www.mgc.ac.cn/cgi-bin/VFs/gene.cgi?GeneID=VFG049149
clbD	4	WP_000982270	http://www.mgc.ac.cn/cgi-bin/VFs/gene.cgi?GeneID=VFG049150
clbE	4	WP_001297917	http://www.mgc.ac.cn/cgi-bin/VFs/gene.cgi?GeneID=VFG049151
clbF	4	WP_000337350	http://www.mgc.ac.cn/cgi-bin/VFs/gene.cgi?GeneID=VFG049152
clbG	4	WP_000159201	http://www.mgc.ac.cn/cgi-bin/VFs/gene.cgi?GeneID=VFG049153
clbH	4	WP_014907257	http://www.mgc.ac.cn/cgi-bin/VFs/gene.cgi?GeneID=VFG049154
clbI	4	WP_000829570	http://www.mgc.ac.cn/cgi-bin/VFs/gene.cgi?GeneID=VFG049155
clbL	4	WP_001297937	http://www.mgc.ac.cn/cgi-bin/VFs/gene.cgi?GeneID=VFG049158
clbM	4	WP_014907260	http://www.mgc.ac.cn/cgi-bin/VFs/gene.cgi?GeneID=VFG049159
clbN	4	WP_001327259	http://www.mgc.ac.cn/cgi-bin/VFs/gene.cgi?GeneID=VFG049160
clbO	4	WP_001029878	http://www.mgc.ac.cn/cgi-bin/VFs/gene.cgi?GeneID=VFG049161
draP	4	AAK16479	http://www.mgc.ac.cn/cgi-bin/VFs/gene.cgi?GeneID=VFG000946
espY1	4	WP_001304001	http://www.mgc.ac.cn/cgi-bin/VFs/gene.cgi?GeneID=VFG034895
focC	4	WP_000975445	http://www.mgc.ac.cn/cgi-bin/VFs/gene.cgi?GeneID=VFG000911
focF	4	WP_000237770	http://www.mgc.ac.cn/cgi-bin/VFs/gene.cgi?GeneID=VFG000913
focI	4	WP_000703304	http://www.mgc.ac.cn/cgi-bin/VFs/gene.cgi?GeneID=VFG000910
gtrA	4	NP_706257	http://www.mgc.ac.cn/cgi-bin/VFs/gene.cgi?GeneID=VFG000669
sfaB	4	WP_000046976	http://www.mgc.ac.cn/cgi-bin/VFs/gene.cgi?GeneID=VFG012329
sfaY	4	WP_000012013	http://www.mgc.ac.cn/cgi-bin/VFs/gene.cgi?GeneID=VFG042389
espY3	3	WP_000978349	http://www.mgc.ac.cn/cgi-bin/VFs/gene.cgi?GeneID=VFG034908
focH	3	WP_001234179	http://www.mgc.ac.cn/cgi-bin/VFs/gene.cgi?GeneID=VFG000915
hlyC	3	WP_000839950	http://www.mgc.ac.cn/cgi-bin/VFs/gene.cgi?GeneID=VFG000842
ibeA	3	AAF98391	http://www.mgc.ac.cn/cgi-bin/VFs/gene.cgi?GeneID=VFG001442
iucD	3	WP_001299620	http://www.mgc.ac.cn/cgi-bin/VFs/gene.cgi?GeneID=VFG000940
iutA	3	WP_016265865	http://www.mgc.ac.cn/cgi-bin/VFs/gene.cgi?GeneID=VFG000936

afaA	2	CAW30797	http://www.mgc.ac.cn/cgi-bin/VFs/gene.cgi?GeneID=VFG042423
afaC_I	2	CAW30799	http://www.mgc.ac.cn/cgi-bin/VFs/gene.cgi?GeneID=VFG042425
afaD	2	CAW30800	http://www.mgc.ac.cn/cgi-bin/VFs/gene.cgi?GeneID=VFG042426
afaF_III	2	AAK16195	http://www.mgc.ac.cn/cgi-bin/VFs/gene.cgi?GeneID=VFG042440
clbJ	2	WP_077265119	http://www.mgc.ac.cn/cgi-bin/VFs/gene.cgi?GeneID=VFG049156
clbK	2	WP_014907259	http://www.mgc.ac.cn/cgi-bin/VFs/gene.cgi?GeneID=VFG049157
espR-	2	WP_000671689	http://www.mgc.ac.cn/cgi-bin/VFs/gene.cgi?GeneID=VFG034815
espY4	2	WP_000225947	http://www.mgc.ac.cn/cgi-bin/VFs/gene.cgi?GeneID=VFG034915
kpsT_1	2	WP_000590258	http://www.mgc.ac.cn/cgi-bin/VFs/gene.cgi?GeneID=VFG050168
mrkJ	2	WP_004149658	http://www.mgc.ac.cn/cgi-bin/VFs/gene.cgi?GeneID=VFG048348
sfaH	2	WP_001234182	http://www.mgc.ac.cn/cgi-bin/VFs/gene.cgi?GeneID=VFG012343
aap_aspU	1	WP_000721278	http://www.mgc.ac.cn/cgi-bin/VFs/gene.cgi?GeneID=VFG034574
afaE_III	1	CAA54121	http://www.mgc.ac.cn/cgi-bin/VFs/gene.cgi?GeneID=VFG001425
aggB	1	AAA57453	http://www.mgc.ac.cn/cgi-bin/VFs/gene.cgi?GeneID=VFG000848
aggC	1	AAA57452	http://www.mgc.ac.cn/cgi-bin/VFs/gene.cgi?GeneID=VFG000849
aggD	1	AAA57451	http://www.mgc.ac.cn/cgi-bin/VFs/gene.cgi?GeneID=VFG000850
espX2	1	WP_010904544	http://www.mgc.ac.cn/cgi-bin/VFs/gene.cgi?GeneID=VFG034859
faeD	1	WP_000761061	http://www.mgc.ac.cn/cgi-bin/VFs/gene.cgi?GeneID=VFG042552
faeI	1	WP_000758128	http://www.mgc.ac.cn/cgi-bin/VFs/gene.cgi?GeneID=VFG042557
faeJ	1	WP_000932014	http://www.mgc.ac.cn/cgi-bin/VFs/gene.cgi?GeneID=VFG042558
focA	1	WP_000768217	http://www.mgc.ac.cn/cgi-bin/VFs/gene.cgi?GeneID=VFG000909
focD	1	WP_001443207	http://www.mgc.ac.cn/cgi-bin/VFs/gene.cgi?GeneID=VFG000912
focG	1	WP_000767894	http://www.mgc.ac.cn/cgi-bin/VFs/gene.cgi?GeneID=VFG000914
icaD	1	WP_000240580	http://www.mgc.ac.cn/cgi-bin/VFs/gene.cgi?GeneID=VFG001286
pic	1	WP_001045652	http://www.mgc.ac.cn/cgi-bin/VFs/gene.cgi?GeneID=VFG000903
sfaS	1	WP_000767895	http://www.mgc.ac.cn/cgi-bin/VFs/gene.cgi?GeneID=VFG012341

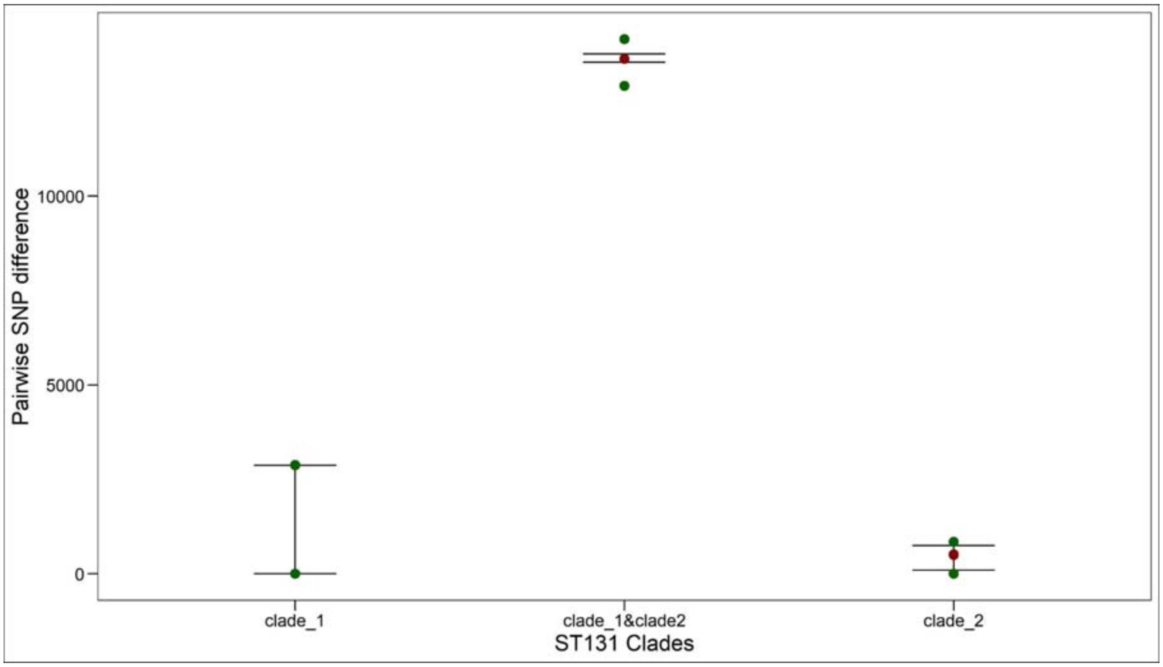

Supplementary Figure 1
Interclade (ST131 clade 1 and ST131 clade 2) distance between lineage ST131 isolates is greater than 10000 SNPs. The "whisker" plot shows the 25th percentile (lower point; green), median (red point), and the 75th percentile (upper point; green) of the pairwise SNP distances within and between lineages.

a) b.)

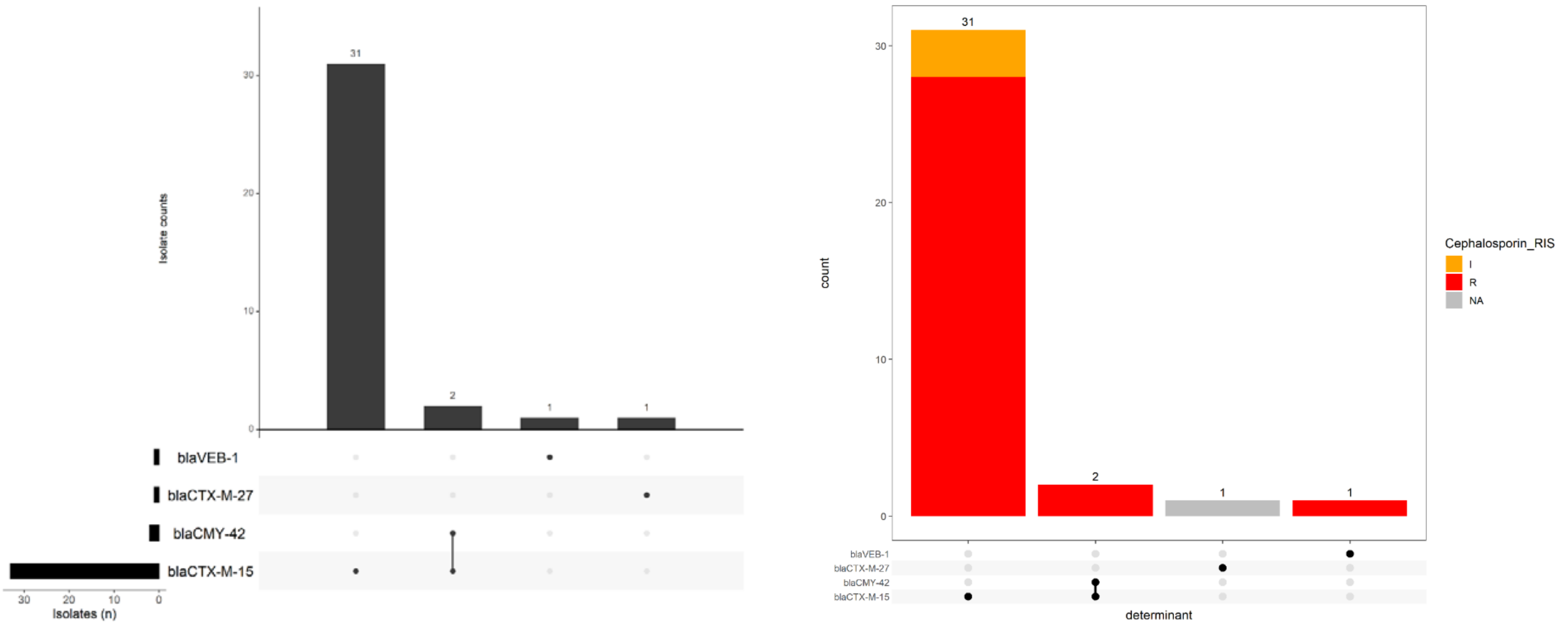

Supplementary Figure. 2: ESBL genes in ExPEC isolates. The upset plots demonstrate (a, b) the number of ExPEC isolates carrying each combination of genes conferring resistance to cephalosporins and (b), is coloured by the proportion of observed phenotypic antimicrobial susceptibility, and is ordered in descending order by the frequency of resistance gene profiles observed. The side bar chart (a) demonstrates the number of isolates that carry each of the resistance genes. The dots and lines between dots at the base of the main bar chart (and the right side of the side bar chart) show the co-resistance gene profile of the ExPEC isolates. From the legend of the coloured upset plot, NA means that there is no available data on phenotypic antimicrobial susceptibility data for the isolate.

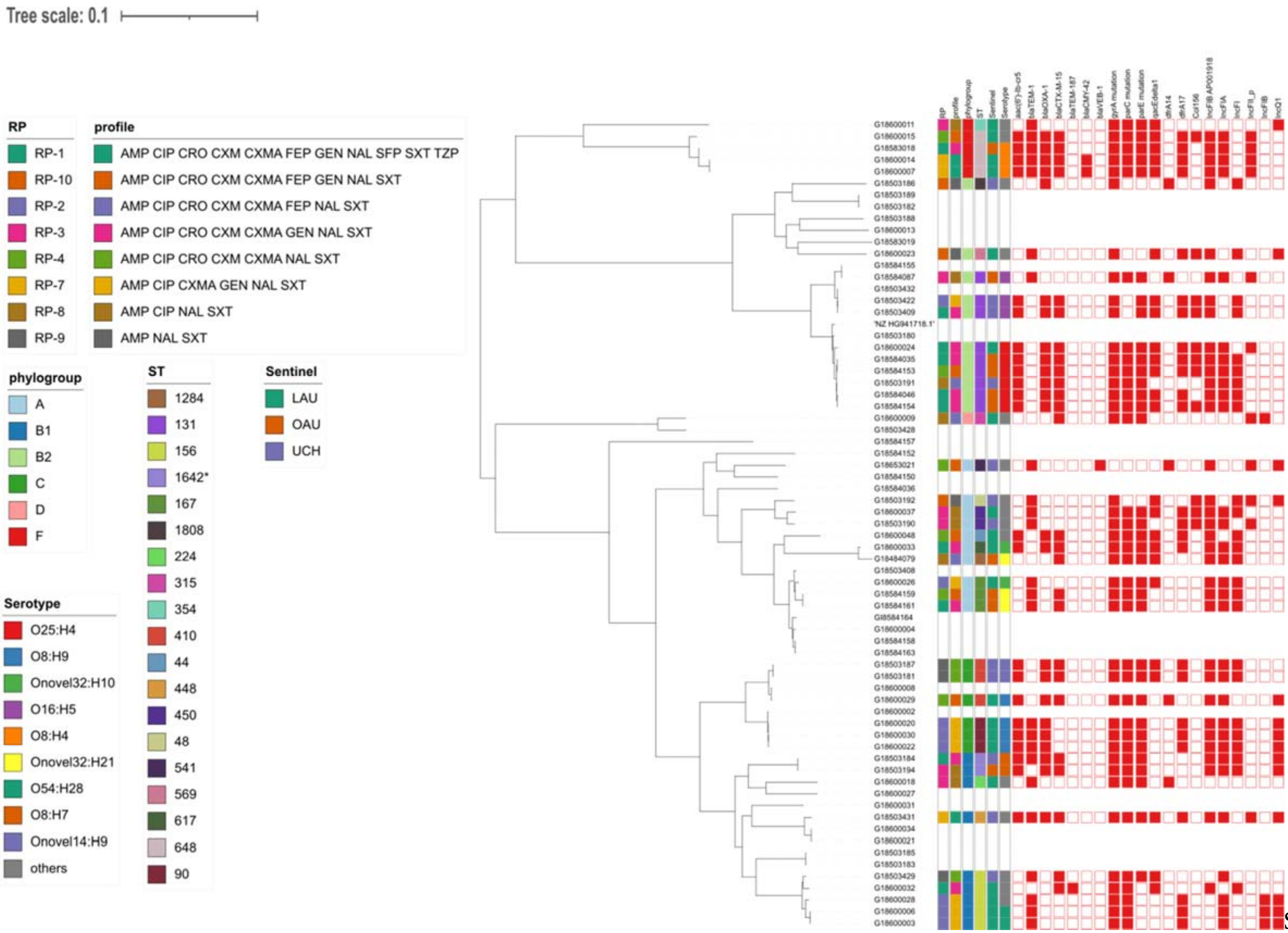

Supplementary Figure 3: Maximum likelihood SNP tree of bloodstream *E. coli* isolates sequenced for this study. Metadata for the 8 most common resistance profiles are

shown in this figure. For the AMR genes and plasmid replicons, red colour indicates presence of the gene.

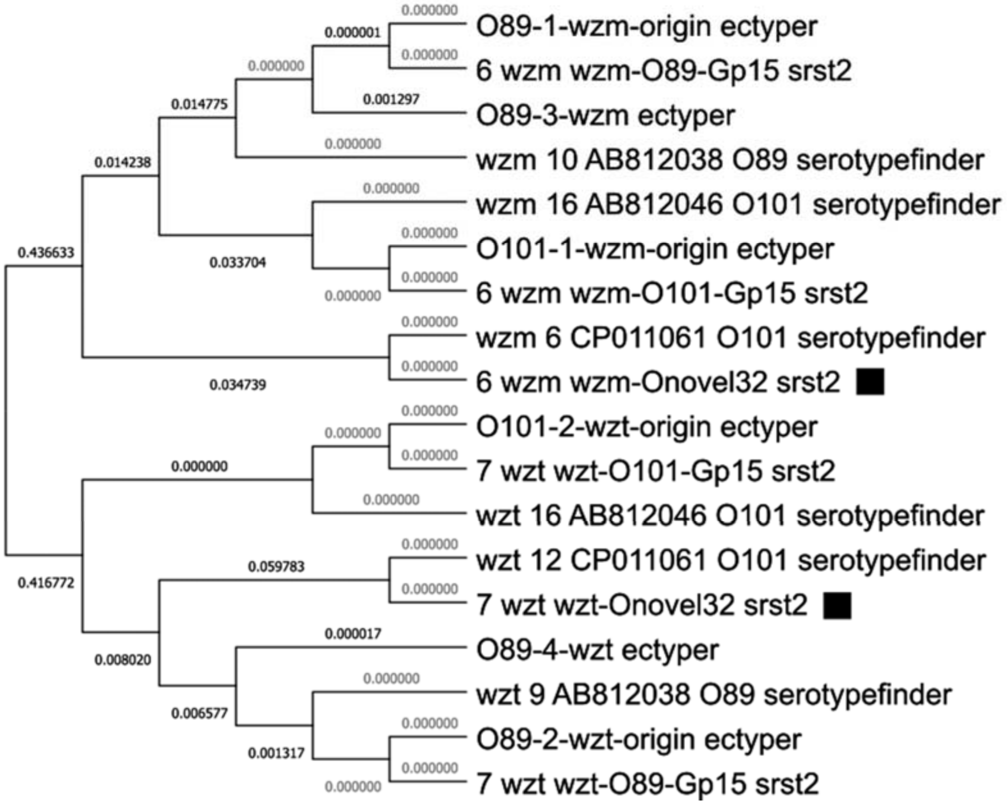

Supplementary Figure 4: Maximum Likelihood Tree showing that Onovel32 gene clusters are evolutionarily related to O101 and O89 gene clusters. The Onovel32 antigen gene clusters are indicated by the black rectangles. The evolutionary history was inferred by using the Maximum Likelihood method and Tamura-Nei model [73]. The tree with the highest log likelihood (-2571.09) is shown. Initial tree(s) for the heuristic search were obtained automatically by applying Neighbor-Join and BioNJ algorithms to a matrix of pairwise distances estimated using the Tamura-Nei model, and then selecting the topology with superior log likelihood value. This analysis involved 18 nucleotide sequences. There were a total of 869 positions in the final dataset. Evolutionary analyses were conducted in MEGA X [35].
